# A new elasmosaurid (Sauropterygia: Plesiosauria) from the non-marine to paralic Dinosaur Park Formation of southern Alberta, Canada

**DOI:** 10.7717/peerj.10720

**Published:** 2021-02-11

**Authors:** James A. Campbell, Mark T. Mitchell, Michael J. Ryan, Jason S. Anderson

**Affiliations:** 1 Department of Biological Sciences, University of Calgary, Calgary, AB, Canada; 2 Royal Tyrrell Museum of Palaeontology, Drumheller, AB, Canada; 3 Department of Earth Sciences, Carleton University, Ottawa, ON, Canada; 4 Department of Comparative Biology and Experimental Medicine, University of Calgary, Calgary, AB, Canada

**Keywords:** Campanian, Dinosaur Park Formation, Elasmosaurid, Lethbridge Coal Zone, Niche-partitioning, Non-marine, Plesiosaur, Fluvial

## Abstract

Elasmosaurid plesiosaurian remains have been documented from non-marine to paralic (fluvial to estuarine) sediments of the upper Campanian Dinosaur Park Formation (DPF) of southern Alberta since 1898. Despite this long collection history, this material has received relatively little research attention, largely due to the highly fragmentary nature of most recovered specimens. However, this assemblage is significant, as it constitutes a rare occurrence of plesiosaurian remains in a non-marine depositional environment. This study reports on a recently collected and prepared specimen, which represents the most complete elasmosaurid yet collected from the DPF. This specimen preserves the trunk region, the base of the neck and tail, a partial fore and hind limb, and tooth, and is sufficiently complete to be assigned as the holotype of a new genus and species. This new taxon is diagnosed by a distinctive character state combination including a boomerang-shaped clavicular arch with acute anterior process, convex anterolateral margin, deeply embayed posterior margin, and pronounced ventral keel, together with the presence of 22 dorsal vertebrae, and the anterior dorsal centra bearing a ventral notch. The DPF plesiosaurian fossils were recovered from both estuarine/bay and fluvial palaeochannel sediments. The holotype skeleton represents an osteologically mature individual with an estimated body length of around 5 m, although the largest referred DPF elasmosaurid might have been closer to 7 m, which is considerably larger than other plesiosaurians reported from non-marine deposits. This suggests small-body lengths relative to typical elasmosaurids from marine settings, but is consistent with other plesiosaurians recovered from non-marine sediments. The identification of a distinct elasmosaurid taxon in the DPF might be evidence of niche-partitioning among the predominantly oceanic members of the ubiquitous plesiosaurian clade.

## Introduction

The Upper Cretaceous (upper Campanian) Dinosaur Park Formation (DPF) is an alluvial to paralic sedimentary unit exposed in southern Alberta and Saskatchewan (Eberth, 2005). These sediments were deposited on the eastern coastal plain of Laramidia, which drained eastwards into the Western Interior Seaway (Blakey, 2020). The DPF has been intensively sampled for over a century (Lambe, 1902), especially in the Dinosaur Provincial Park (DPP) region of southern Alberta and has yielded a highly diverse assemblage of fossil vertebrates including hybodontiform and rhinobatoid chondrichthyans and acipenseriform, holostean, and teleost osteichthyans (Neuman & Brinkman, 2005), lissamphibians (Gardner, 2005), adocid, baenid, chelydrid, macrobaenid, nanhsiungchelyid, and trionychid turtles (Brinkman, 2005), choristoderans (Gao & Brinkman, 2005), helodermatid, mosasaurid, necrosaurid, varanid, and xenosaurid squamates (Caldwell, 2005), elasmosaurid and polycotylid plesiosaurians (Sato et al., 2005), alligatoroid crocodylians (Wu, 2005), azhdarchid pterosaurs (Godfrey & Currie, 2005), ankylosaurian, ceratopsian, ornithopod, pachycephalosaurian, and theropod dinosaurs (Ryan & Evans, 2005; Campbell et al., 2016; McFeeters et al., 2016), and marsupial, multituberculate, and placental mammals (Fox, 2005).

The plesiosaurian remains from the DPF were first collected in 1898 and recognized by Lambe (1902) from exposures within DPP, and his description of these fossils was the first ever made for an elasmosaurid from Canada (Christison, Tanke & Mallon, 2020). However, the earliest-known collection of elasmosaurid fossils from Canada was made in 1881 in southeastern Alberta and likely derived from the lower Campanian Eagle Sandstone Formation (Christison, Tanke & Mallon, 2020). Despite the fact that this material represented a rare example of plesiosaurian remains from non-marine sediments (see Supplemental File 1 for summary list of non-marine plesiosaurian occurrences), it received relatively little research attention for over a century. Sato et al. (2005) conducted a thorough taxonomic, anatomical, and stratigraphic survey of plesiosaurian material collected from the DPF. They identified almost all of the fossils as pertaining to elasmosaurids, and some teeth as possibly referable to polycotylids; collectively these stratigraphically span most of the DPF succession. Sato et al. (2005) further recognized that the DPF plesiosaurian remains typically occurred as isolated elements in multitaxic bonebeds that formed as lag deposits in high-energy palaeochannels. Rare, associated partial skeletons were also found in palaeochannels associated with low-energy point-bar deposits (Sato et al., 2005).

Based on their relative degrees of ossification, Sato et al. (2005) tentatively identified the DPF plesiosaurian specimens as comprising both osteologically immature (“juvenile”) and mature (“adult”) individuals. Perhaps most remarkably, however, they noted that the DPF assemblage consisted of elements that were small relative to those of elasmosaurids found in more offshore, marine deposits such as the Bearpaw or Pierre Shale formations, with larger elements conspicuously absent in the DPF. Incomplete preservation has, however, hitherto hindered body size estimations and taxonomic assignments.

This study describes an elasmosaurid skeleton (TMP 2009.037.0068/1990.046.0001/.0002) collected from the DPF in southeastern Alberta between 1990 and 2012. This specimen is the most complete example of an elasmosaurid yet known from the DPF, and is designated the holotype of a new genus and species herein. We also describe a second referred specimen (TMP 2009.037.0007) that was found near the holotype but represents a proportionately larger conspecific individual.

## Geological setting

The Belly River Group is a predominantly terrestrial sedimentary sequence deposited along the western margin of the Western Interior Seaway (WIS; Eberth, 2005). The WIS was a large, shallow marine corridor that extended from what is now the Arctic Ocean to the Gulf of Mexico, and divided North America into the microcontinents of Appalachia in the east and Laramidia in the west (Blakey, 2020). The Belly River Group includes the Foremost, Oldman, and Dinosaur Park formations in ascending stratigraphic order (Eberth, 2005).

**Figure 1:**
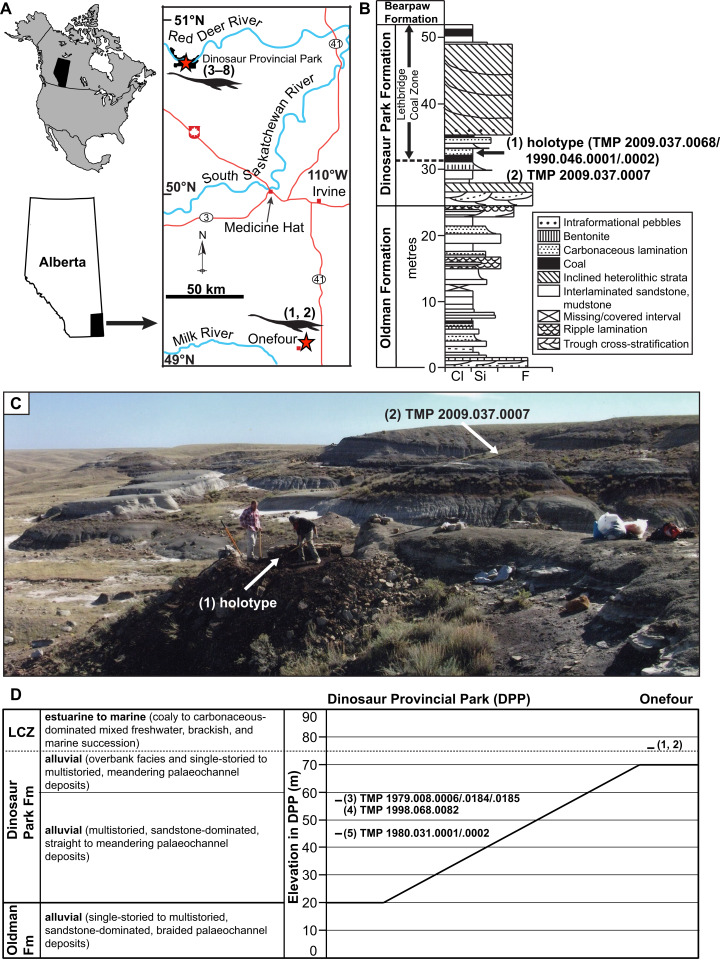
Geographic locality and stratigraphic position of specimens of new genus and species of elasmosaurid. Holotype TMP 2009.037.0068/1990.046.0001/.0002 (1) and referred specimens TMP 2009.037.0007 (2), TMP 1979.008.0006/.0184/.0185 (3), TMP 1998.068.0082 (4), TMP 1980.031.0001/.0002 (5), CMN 304–309/312–314 (6), CMN 9895 (7), and CMN 51829 (8). (A) Locality of specimens in southern Alberta (red stars); (B) position of holotype and TMP 2009.037.0007 within the Dinosaur Park Formation (DPF; stratigraphic section redrawn and modified from Eberth & Hamblin (1993: fig. a5); grain size abbreviations – cl = clay, si = silt, f = fine sand); (C) photograph of holotype quarry during excavation, with pedestaled specimen indicated by arrow (photograph courtesy of the TMP), and approximate area where TMP 2009.037.0007 was collected (arrow); and (D) generalized stratigraphic relationships of the DPF and Oldman Formation between DPP and Onefour, Alberta, their depositional environments, and stratigraphic positions of specimens (modified from Eberth & Hamblin (1993: fig. 19a) and Eberth (2005: fig. 3.1)). Note that the holotype and TMP 2009.037.0007 were collected from an estuarine environment, whereas TMP 1979.008.0006/.0184/.0185, TMP 1998.068.0082, and TMP 1980.031.0001/.0002 were collected from fluvial palaeochannel deposits.

The DPF was deposited during the last major transgression of the WIS, and transitions from a lower alluvial sandy unit with meandering palaeochannels, to an upper alluvial muddy unit dominated by overbank deposits that are finally overlain by the Lethbridge Coal Zone (LCZ; Fig. 1D; Eberth, 2005). The DPF is capped by marine shales of the Bearpaw Formation. The LCZ consists of coal beds less than 1 m thick, as well as U-shaped mudstone-filled incised valleys distributed along a wave-dominated shoreline (Eberth, 2005). The lower two thirds of the DPF, as exposed in DPP, are estimated to have been deposited between 250 and 100 km west of the WIS shoreline (Eberth, 2005). Palaeochannel reconstructions in the DPF range from 35 to 165 m, and possibly up to >200 m in width, and between 5 and 25 m in maximum water depth (Wood, 1989; Eberth, 2005).

The uppermost 20 m of the Oldman Formation, together with the entire 70 m of the DPF, and lowermost 20 m of the Bearpaw Formation are exposed in the DPP area (Fig. 1D; Eberth, 2005). The Oldman Formation and DPF both thicken towards their respective sediment sources along the rising Cordillera to the west. The contact between these units is diachronous, becoming younger towards the south and east of DPP (Eberth & Hamblin, 1993). As a result, the DPF is only 30 m thick in the Onefour area of southeasternmost Alberta, and the uppermost sediments of the Oldman Formation in that region are coeval with the DPF in DPP (Fig. 1D).

TMP 2009.037.0068/1990.046.0001/.0002 was initially discovered by Donna Sloan (Royal Tyrrell Museum of Palaeontology, Drumheller, AB, Canada) on the Sage Creek Provincial Grazing Reserve near Onefour (Fig. 1; precise locality data on file at the TMP). In 1990, she collected some closely associated in-situ and ex-situ elasmosaurid elements laying within a 1.5 m^2^ area. These were catalogued as TMP 1990.046.0001 and include two dorsal, one sacral, and two caudal vertebrae, two sacral ribs, one gastralium, and a few rib fragments. TMP 1990.046.0002 otherwise comprises a cervical vertebra and phalanx, but additional bones were reportedly still in-situ. Wendy Sloboda subsequently returned to the site in 2009 as part of the Southern Alberta Dinosaur Project field season. An associated, partial skeleton (TMP 2009.037.0068) from this site was subsequently uncovered in 2010–2011, and fully excavated in 2012 by TMP staff. In 2009, Wendy Sloboda discovered a second locality approximately 150 m away that yielded TMP 2009.037.0007. This specimen comprised a humerus, rib, and gastralium, which were collected in 2010 by TMP staff. These fossils derive from strata immediately overlying a coal seam, associated with a decimetre thick, carbonaceous sandstone bed containing abundant plant material. The coal seam is the lowest coal bed exposed in the area, and is interpreted as the base of the LCZ (Fig. 1).

TMP 2009.037.0068, TMP 1990.046.0001, and TMP 1990.046.0001 probably represent a single individual because they derive from the same site and are indistinguishable in size, colour, bone texture and degree of osteological maturity. The vertebrae of TMP 1990.046.0001 also sequentially fill gaps along the column of TMP 2009.037.0068. We refer to this individual as the composite ‘holotype’ herein for convenience.

Two sediment samples associated with the holotype were analyzed by D. Braman (TMP) in 2016. Three dinoflagellate specimens were identified, suggesting a marine-influenced depositional environment. However, both the diversity and abundance of this assemblage are exceptionally low relative to those typically seen in open marine sediments. This suggests a more restricted marginal marine setting, such as an estuary or bay (D. Braman, 2017, personal communication). Other vertebrates found associated with the holotype include the chelonioid *Kimurachelys slobodae* (Brinkman et al., 2015) and the rhinobatoid ray *Myledaphus*. Remains of *Kimurachelys* are also known from nearshore sediments in the LCZ (Brinkman et al., 2015), and *Myledaphus* is common throughout the non-marine sequences of the DPF (Neuman & Brinkman, 2005).

**Figure 2:**
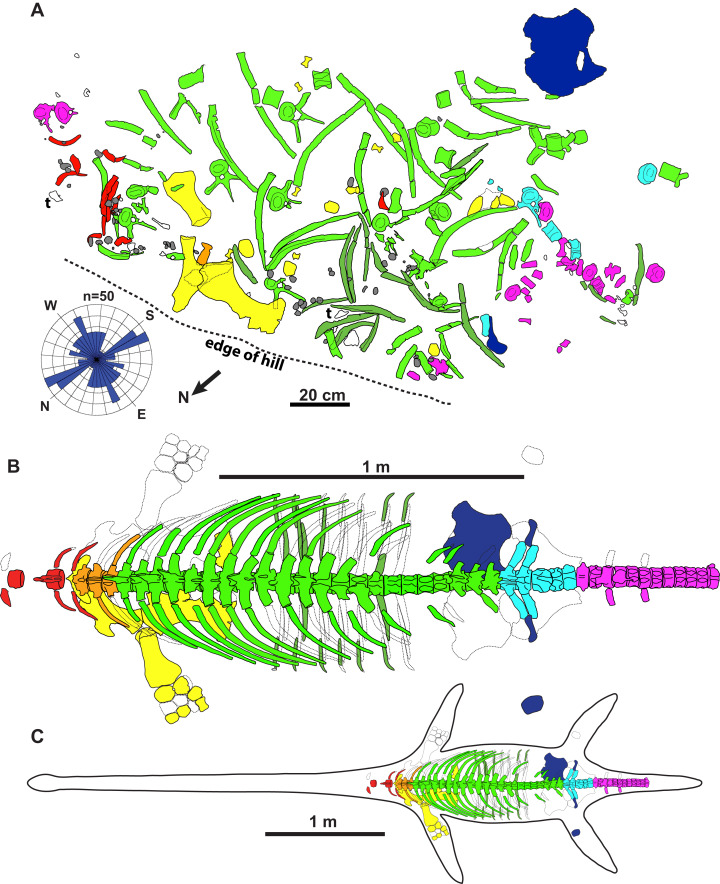
Quarry map and reconstruction of holotype of new genus and species of elasmosaurid. Map (A) is of jacketed specimen and is upside down in relation to how the specimen was found in the field. Rose diagram of elongate skeletal elements (*n* = 50) shown in bottom left. Grey = gastroliths, “t” = turtle shell fragments, white = unidentifiable elements. The elements had a non-significant mean resultant trend of 92.3°/272.3° (A); 95% confidence interval for mean = 347.6°–197.0°; circular variance = 0.473; Rayleigh Test–*Z* = 0.144, *p* = 0.866; Rao’s Spacing Test–*U* = 134.941, 0.50 > *p* > 0.10; Watson’s *U*2 Test–*U*2 = 0.053, *p* > 0.5; Kuiper’s Test–*V* = 1.073, *p* > 0.15. Dorsal view of specimen (B) and specimen with estimated body outline (C).

A photomap of the jacketed block containing the holotype was assembled in the preparatory lab at the TMP, and shows the side of the specimen that lay face-down (B. Sanchez, 2016, personal communication; Fig. 2A); the compass rose is flipped accordingly in Fig. 2A. The northeastern margin of this block was discovered eroding out of the top of a hill, with some additional elements found ex-situ in the immediate vicinity. These included vertebrae, ribs, gastralia, parts of the pectoral girdle, and phalanges catalogued under TMP 1990.046.0001, TMP 1990.046.0002 and TMP 2009.037.0068 (not shown in Fig. 2A). The quarry was expanded laterally (southwestwards) into the hill, which yielded a few additional bones (not shown in Fig. 2A). Most of the skeleton was subsequently collected as a large, jacketed block, which was removed by helicopter. Both the holotype and TMP 2009.037.0007 were prepared at the TMP in 2012–2013.

## Materials and Methods

The sites which yielded the specimens TMP 2009.037.0068 and TMP 2009.037.0007 were accessed by a Research and Collection Permit (File No. 3951-E03; provided by Alberta Culture and Community Spirit Heritage Division and the Royal Tyrrell Museum of Palaeontology, Drumheller, AB, Canada) issued to David Evans (Royal Ontario Museum, Toronto, ON, Canada). Photographs were taken using a Canon E03 Rebel T5i digital SLR camera with an 18–55 lens and 180 mm ultrasonic Macrolens. Image manipulation was performed in Adobe Photoshop CS5.1. Figures were prepared in Adobe Illustrator CS5.1. Measurements were taken to the nearest mm using digital calipers for measurements up to 300 mm. Measurements of skeletal elements and gastroliths are provided in Supplemental File 2.

The electronic version of this article in Portable Document Format (PDF) will represent a published work according to the International Commission on Zoological Nomenclature (ICZN), and hence the new names contained in the electronic version are effectively published under that Code from the electronic edition alone. This published work and the nomenclatural acts it contains have been registered in ZooBank, the online registration system for the ICZN. The ZooBank Life Science Identifiers (LSIDs) can be resolved and the associated information viewed through any standard web browser by appending the LSID to the prefix http://zoobank.org/. The LSID for this publication is: urn:lsid:zoobank.org:pub:705EFEB6-07D0-4A22-8614-AD436D8AE8DD. The online version of this work is archived and available from the following digital repositories: PeerJ, PubMed Central and CLOCKSS.

## Results

### Systematic Palaeontology

Sauropterygia Owen, 1860

Plesiosauria De Blainville, 1835

Elasmosauridae Cope, 1869

*Fluvionectes* gen. nov.

urn:lsid:zoobank.org:act:04CEAEEA-C706-478E-BD51-F802C4DAF746

*Fluvionectes sloanae* sp. nov.

urn:lsid:zoobank.org:act:A7D6D773-1329-40EB-9EE4-C51C96955AD4

**Holotype**

TMP 2009.037.0068/1990.046.0001/.0002, partial skeleton consisting of a tooth, posterior cervical vertebral series, the complete pectoral, dorsal, and sacral vertebral series, the anterior half of the caudal vertebral series, ribs, gastralia, partial pectoral and pelvic girdles, and a partial fore and hind limb.

**Type locality and horizon**

Sage Creek Provincial Grazing Reserve, near Onefour, Alberta. Precise locality data on file at the TMP. TMP 1990.046.0001, TMP 1990.046.0002, and TMP 2009.037.0068 all derive from a stratum immediately overlying the basalmost coal bed of the Lethbridge Coal Zone in the Dinosaur Park Formation (DPF), upper Campanian, Upper Cretaceous.

**Etymology**

The genus name is derived from “fluvius”, the Latin word for river, and “nectes”, the Latinized Greek word (nektes) for swimmer (gender; masculine). We are aware that the connecting vowel “o” is inappropriate, but this is a deliberate choice on our part as we prefer this spelling and pronunciation. This is not an inadvertent error and, therefore, does not require subsequent correction according to the International Code of Zoological Nomenclature (International Commission on Zoological Nomenclature, 1999: art. 32.5). The species name honours Donna Sloan who discovered the holotype, and for her long service to palaeontology, both in the field and as the scientific illustrator at the Royal Tyrrell Museum of Palaeontology (gender; feminine).

**Diagnosis**

Elasmosaurid possessing a boomerang-shaped clavicular arch with an acute anterior process, convex anterolateral margin, posterior margin with a deep embayment on either side, and a pronounced ventral keel, 22 dorsal vertebrae, and anterior dorsal centra with a ventral notch. Other manifest character states include: posterior cervical vertebra with high dorsoventral aspect; three pectoral vertebrae; five sacral vertebrae; scapula with an elongate dorsal ramus; coracoid with an open (non-enclosed) cordiform intercoracoid fenestra; pubis with an anterolateral embayment; and a postaxial supernumerary epipodial facet on the humerus.

**Referred specimens**

CMN 304–309/312–314, six cervical and five dorsal vertebrae, both humeri, epipodials, mesopodials, phalanges, and other fragments. CMN 9895, a right pubis and partial right ischium. CMN 51829 (previously referred to by Sato et al., 2005 as: CMN “Lambe numbers” 475r-z, ww, xx, yy, CMN 1079), 11 cervical, one dorsal, and one pectoral (or sacral) vertebrae. TMP 1979.008.0006/.0184/.0185, dorsal vertebra and both pubes. TMP 1980.031.0001/.0002, six cervical and 10 dorsal vertebrae, ribs, one partial scapula and coracoid, ilium and limb elements. TMP 1998.068.0082, two dorsal vertebrae. TMP 2009.037.0007, partial rib and gastralium, and left humerus.

**Locality and horizon of referred specimens**

TMP 1979.008.0006/.0184/.0185 and TMP 1998.068.0082 were both collected in DPP, Alberta, 37 m above the base of the DPF (in 1979 and 1998, respectively). CMN 304–309/312–314, CMN 9895, and CMN 51829 were also collected from the DPF of DPP (in 1913, 1921 and 1898, respectively), although their precise localities are unknown. TMP 2009.037.0007 was collected in 2010 from the same horizon as the holotype.

**Taphonomy of the holotype**

The holotype skeleton of *Fluvionectes sloanae* was found disarticulated and dispersed over an area of approximately 2.5 m^2^ (Fig. 2A). The only elements remaining in articulation were two lateral gastralia. Several large fragments of petrified wood were found to the immediate southwest and may represent a log jam. The torso otherwise appears to have been fully articulated when it came to rest on the substrate, but underwent considerable lateral displacement prior to burial, possibly via the actions of scavengers (although there are no obvious bite traces or other pathological bone modifications), wave action, and/or bottom currents. The left scapula, coracoid, and humerus are among the largest preserved skeletal elements, and were found in close association. The coracoid, humerus, and two articulated gastralia were found ventral-side down, suggesting that the carcass was dorsal-side up. Nonetheless, the pubis was found ventral-side up and distanced from the other bones, suggesting some hydraulic transport. The head, neck, distal appendicular elements, and posterior half of the tail are all missing, and were perhaps detached during bloat-and-float decomposition (Barnes & Hiller, 2010). Other plesiosaurian remains from the DPF are similarly often found in a disarticulated state (Sato et al., 2005).

To determine whether the elements have a preferred orientation, possibly due to current action, the orientations of mapped elements (*n* = 50) at least twice as long than wide and measuring at least 100 mm were taken from Fig. 2A; the two articulated gastralia were treated as a single unit. A circular histogram (rose diagram) and axial statistics—including one-sample Rayleigh, Rao’s Spacing, Watson’s *U*2 and Kuiper’s tests—of these data were plotted and run, respectively, using the software Oriana v.4 a circular statistics program by Kovach Computing Services (2018).

The elements also show no evidence of plastic deformation due to diagenesis, although most are extensively fractured and have suffered from erosion damage prior to discovery. Some ex-situ elements were also found adjacent to the quarry, including some vertebrae, ribs, gastralia, parts of the pectoral girdle, and phalanges catalogued under TMP 1990.046.0001, TMP 1990.046.0002 and TMP 2009.037.0068 (not shown in Fig. 2A).

**Description of the holotype**

**Dentition**

**Figure 3:**
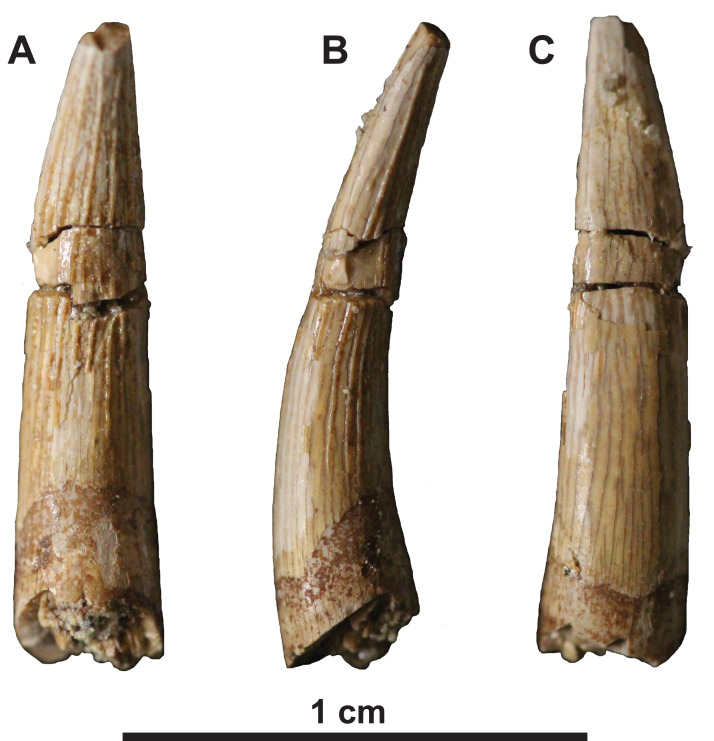
Tooth of holotype of *Fluvionectes sloanae*, gen. et sp. nov. (A) Lingual, (B) profile and (C) labial views.

The isolated tooth lacks both its root and the crown tip (Fig. 3). It is slender and lingually curved in form, with slight labiolingual compression, which resembles the teeth of other elasmosaurids, but differs from the tooth crowns of polycotylids, which are more conical in shape (Druckenmiller & Russell, 2008b; Kear et al., 2017). The enamel is thin and cracked, but the lingual and lateral surfaces have distinct ridges that continue to the apex, whereas the labial surface is smooth like those of other elasmosaurids (Kear et al., 2017).

**Axial skeleton**

**Figure 4:**
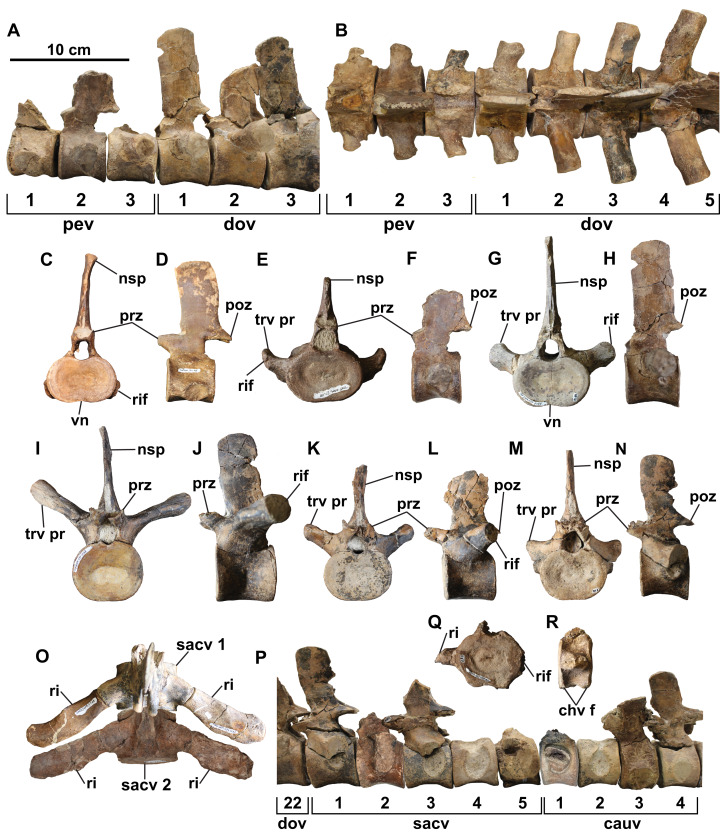
Select vertebrae of holotype of *Fluvionectes sloanae*, gen. et sp. nov. Articulated pectoral and anterior dorsal vertebrae in: (A) left lateral and (B) dorsal views. Cervical vertebra in: (C) anterior and (D) left lateral views. Pectoral vertebra 2 in: (E) anterior and (F) left lateral views. Dorsal vertebra 1 in: (G) anterior and (H) left lateral views. Dorsal vertebra 11 in: (I) anterior and (J) left lateral views. Dorsal vertebra 20 in: (K) anterior and (L) left lateral views. Sacral vertebra 1 in: (M) anterior and (N) left lateral views. Sacral vertebrae 1 and 2 and associated ribs in (O) dorsal view. Articulated posterior dorsal, sacral, and anterior caudal vertebrae in (P) left lateral view (sacral vertebra 2 and caudal vertebra 1 flipped). Caudal vertebra 12 in: (Q) anterior and (R) right lateral views. See “Anatomical Abbreviations”.

A total of 44 platycoelous vertebrae are preserved, including two posterior cervical, three pectoral, 22 dorsal, five sacral and 12 anterior caudal vertebrae (Figs. 2B and 4). These counts are consistent with those of other elasmosaurid taxa, and, except for the cervical vertebrae, appear to represent a continuous series since there are no major discrepancies in size or morphology. The vertebrae are numbered sequentially herein, based on their probable anatomical order. Ribs are not attached to any of the vertebrae, except for one cervical and two caudal vertebrae. The neurocentral sutures are fused and closed externally in many of the vertebrae. In all vertebrae, where preserved, the zygapophyses are horizontal in lateral view and separated by a median slit; except for the prezygapophyses of the cervical vertebrae, which are conjoined along their entire length. The prezygapophyses are also angled ventromedially to form a ‘V-shape’ in articular view. Their combined width is distinctly narrower than the centrum.

**Cervical vertebrae:** The two cervical vertebrae are identified by their rib facets being situated entirely on the body of the centrum. They also bear median ventral notches, which are visible in anterior view (Figs. 4C and 4D), and are characteristic of most elasmosaurids except for *Callawayasaurus* (Welles, 1943), *Eromangasaurus* (Kear, 2005, 2007), and *Lagenanectes* (Sachs, Hornung & Kear, 2017). The best-preserved cervical vertebra appears to be the second-to-last in the series. The other, less complete cervical vertebra is similar to the first, except it is slightly smaller and would have been situated slightly more anteriorly; the proximal end of a rib is also fused with this centrum.

**Figure 5:**
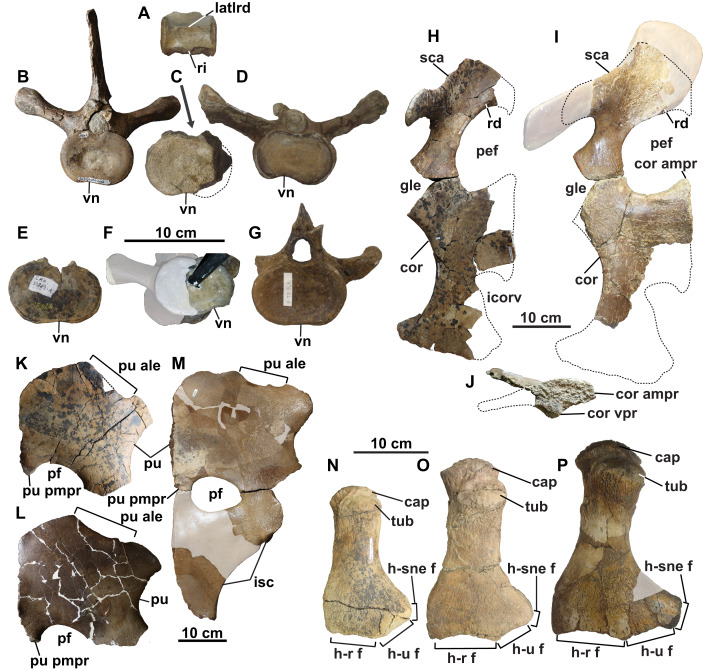
Vertebrae, pectoral and pelvic girdles, and humeri of *Fluvionectes sloanae*, gen. et sp. nov. from the Dinosaur Park Formation. (A) Cervical vertebra of CMN 304–309/312–314 (referred specimen) in lateral view; (B) dorsal vertebra (dorsal vertebra 4) of holotype in anterior view; (C) dorsal vertebra of CMN 304–309/312–314 (referred specimen) in anterior or posterior view; (D) dorsal vertebra of TMP 1998.068.0082 (referred specimen) in anterior view; (E) dorsal vertebra of CMN 51829 (referred specimen) in anterior or posterior view; (F) dorsal vertebra of TMP 1980.031.0001/.0002 (referred specimen) in posterolateral view; (G) dorsal vertebra of TMP 1979.008.0006/.0184/.0185 (referred specimen) in anterior view; (H) left scapula and coracoid of holotype in dorsal view; (I) left scapula and coracoid of TMP 1980.031.0001/.0002 (referred specimen) in dorsal view; (J) left coracoid of TMP 1980.031.0001/.0002 (referred specimen) in medial view; (K) pubis of holotype in ventral view (flipped); (L) left pubis of TMP 1979.008.0006/.0184/.0185 (referred specimen) in ventral view; (M) right pubis and ischium of CMN 9895 (referred specimen) in dorsal view; (N) left humerus of holotype in dorsal view; (O) left humerus of TMP 2009.037.0007 (referred specimen) in dorsal view; and (P) right humerus of CMN 304–309/312–314 (referred specimen) in dorsal view (flipped). Note lateral longitudinal ridge on cervical vertebra (A), ventral notch on dorsal vertebrae (B–G), anterolateral embayment on pubes (K–M), and supernumerary epipodial facet on humeri (N–P). Grey areas in (F), (I), (M) and (P) represent reconstructed regions. Dotted lines in (C) and (H)–(K) denote estimated reconstructed margin. See “Anatomical Abbreviations”.

The cervical centrum is wider than long, and longer than high, as is typical of elasmosaurid posterior cervical vertebrae, which typically decrease in relative length progressing both anteriorly and posteriorly away from the middle cervical vertebral region (O’Keefe & Hiller, 2006; Sachs & Kear, 2015). There appears to be a gap in the sequence between the cervical vertebrae and first pectoral vertebra based on their distinct difference in width, and the ventral positioning of the cervical rib facet (this is instead placed approximately halfway up the centrum in the posteriormost cervical vertebra; Sachs, Kear & Everhart, 2013). The centrum is slightly dorsoventrally constricted between the articular ends (Fig. 4D). The rib facet of the cervical vertebra is gently concave, oval-shaped, posteriorly off-centre, and angled posteriorly. Two nutrient foramina (foramina subcentralia) are present on the ventral surface of the cervical vertebra. There is no lateral longitudinal ridge, which is otherwise present on elasmosaurid cervical vertebrae except for the posteriormost ones (Welles, 1943; Kubo, Mitchell & Henderson, 2012; O’Gorman et al., 2015). Lateral longitudinal ridges are absent in the cervical vertebrae of *Kaiwhekea* (Cruickshank & Fordyce, 2002) and *Nakonanectes* (Serratos, Druckenmiller & Benson, 2017). However, lateral longitudinal ridges are present in the referred specimen CMN 304–309/312–314, which preserves more anteriorly-situated cervical vertebrae (Fig. 5A).

The neural arch is fused to the centrum, but the neurocentral suture can be discerned. The pedicles of the neural arch are mediolaterally-broad anteriorly and tapered posteriorly. In lateral view, the anteroventral margin slopes steeply posteroventrally, but the posteroventral margin rises posterodorsally at a gentler angle. The high neural spine is about 1.5 times higher than the centrum, which is consistent with the posterior cervical vertebrae of most elasmosaurids except for *Aristonectes quiriquinensis* (Otero et al., 2014). The neural spine is also rectangular in lateral view, gently anteriorly inclined, and mediolaterally narrow. Its dorsal end is thickened to a greater degree than in the remaining post-cervical vertebrae.

**Pectoral vertebrae:** The three pectoral vertebrae (following the definition proposed by Sachs, Kear & Everhart, 2013) are intermediate in size between the cervical and dorsal vertebral series, and bear a rib facet that extends across both the neural arch and centrum (Figs. 4A, 4B, 4E, and 4F). The diapophysis progressively contributes a greater proportion of the rib facet posteriorly along the series. The rib facet is angled posteriorly. A count of three pectoral vertebrae is consistent with most other elasmosaurids, although some have as few as two (*Hydrotherosaurus*, Welles, 1943; *Libonectes*, Sachs & Kear, 2017; *Morenosaurus*, Welles, 1943), and others as many as five (*Callawayasaurus*; Welles, 1962; Benson & Druckenmiller, 2014). The pectoral centra are wider than tall, with the length of pectoral vertebrae 1 and 2 being comparable to their height; pectoral vertebra 3 is instead longer than high. Pectoral vertebrae 1 and 2 have a flat ventral margin, but pectoral 3 has a faint ventral notch. Paired foramina subcentralia are present on each centrum. None of the pectoral vertebrae preserve a complete neural spine.

**Dorsal vertebrae:** The 22 dorsal centra lack rib facets, which are instead borne on the transverse processes; where preserved these are circular to oval in shape (Figs. 4A, 4B and 4G–4L). The dorsal vertebral count exceeds that of most elasmosaurids, including *Hydrotherosaurus* (15; Welles, 1943), *Kawanectes* (15; O’Gorman, 2016), *Albertonectes* (16; Kubo, Mitchell & Henderson, 2012; Sachs, Kear & Everhart, 2013), *Morenosaurus* (17; Welles, 1943), *Vegasaurus* (17; O’Gorman et al., 2015), *Futabasaurus* (18; Sato, Hasegawa & Manabe, 2006), CM Zfr 115 (18; Hiller et al., 2005, 2017) and *Kaiwhekea* (19 or 20; Cruickshank & Fordyce, 2002), but is less than that of *Callawayasaurus* (23; Welles, 1962) and *Thalassomedon* (25; Welles, 1943). There is a variably-developed ventral notch on centra 1–8 and 22 (Figs. 4G and 4H), while the other centra have flat ventral margins (Figs. 4I–4L). Each centrum has two or more foramina subcentralia. The transverse processes are angled posteriorly, as are the rib facets, with the longest and most steeply inclined being in the middle dorsal vertebral region; the facets are otherwise shorter and less inclined both anteriorly and posteriorly along the column. The neural spine is tall and vertical in lateral view.

Ventral notches have not been previously reported in plesiosaurian dorsal vertebrae. Wiffen & Moisley (1986: figs. 25, 26, 28 and 29) figured two elasmosaurid centra from New Zealand with a notch (NZGS CD428 and NZGS CD429) and described them as being dorsal centra. However, NZGS CD428 is most likely a cervical centrum, as it appears to preserve rib facets (J. O’Gorman, 2020, personal observation). The centrum of NZGS CD429, as figured by Wiffen & Moisley (1986: figs. 28 and 29), is actually a chimaera of an isolated cervical centrum with rib facets and an isolated dorsal vertebral neural arch. Sato, Hasegawa & Manabe (2006) considered both of these specimens to be indeterminate elasmosaurids. Dorsal centra with ventral notches are also present in several other elasmosaurid specimens from the DPF, including CMN 304–309/312–314 (Fig. 5C; considered by Sato et al. (2005) as all belonging to the same individual), TMP 1998.068.0082 (Fig. 5D; misidentified as a cervical vertebra by Sato et al., 2005: fig. 14.4g), CMN 51829 (Fig. 5E), TMP 1980.031.0001/.0002 (Fig. 5F), and TMP 1979.008.0006/.0184/.0185 (Fig. 5G). All of these specimens are considered to be conspecific with the holotype of *Fluvionectes sloanae*.

**Sacral vertebrae:** The five sacral vertebrae are defined by their rib facets being shared between the centrum and transverse process, but with rib facets that are larger than those of the pectoral vertebrae (Figs. 4M–4P). The centrum contributes a greater proportion of the rib facet along the sacral vertebral series. The rib facets are angled posteriorly. A count of five sacral vertebrae compares with *Albertonectes* (Kubo, Mitchell & Henderson, 2012) and *Hydrotherosaurus* (Welles, 1943), but exceeds that of many documented elasmosaurids, including *Libonectes* (two; Sachs & Kear, 2017), *Styxosaurus* sp. –‘*Hydralmosaurus serpentinus*’ holotype (three; Otero, 2016), CM Zfr 115 (three; Hiller et al., 2005, 2017), *Morenosaurus* (three; Welles, 1943), *Thalassomedon* (three; Welles, 1943), *Vegasaurus* (three; O’Gorman et al., 2015), *Kawanectes* (possibly three; O’Gorman, 2016), *Kaiwhekea*, (either three or four; Cruickshank & Fordyce, 2002), *Elasmosaurus* (four; Sachs, 2005) and *Terminonatator* (at least four; Sato, 2003). The number of foramina subcentralia on each sacral centrum ranges from one to four. The neural spines of the sacral vertebrae are fairly vertical in lateral view.

**Caudal vertebrae:** The 12 caudal vertebrae have rib facets situated entirely on the centrum, but their centra are more circular in articular view and most bear chevron facets on the ventral margin (Figs. 4P–4R). The rib facets are variably angled laterally to posteriorly. The three largest and anteriormost caudal vertebrae lack chevron facets and are transitional between the sacral and more posterior caudal vertebrae. The chevron facets first appear on caudal vertebra 10, and progressively migrate from the posterior end of the centrum to a position between adjacent centra along the column, resulting in a facet on both the anterior and posterior margins of the centrum. The relative length of the centrum decreases posteriorly along the caudal vertebral series. The number of foramina subcentralia on each caudal vertebra varies from one to two. The neural spines of the caudal vertebrae are vertical in lateral view.

**Figure 6:**
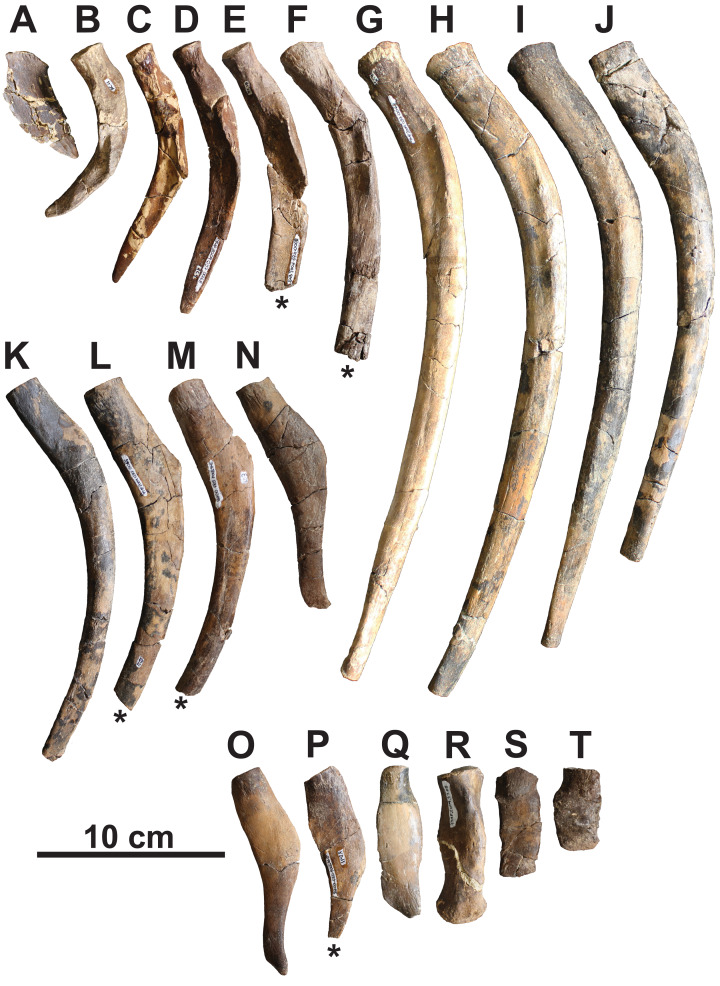
Representative ribs of holotype of *Fluvionectes sloanae*, gen. et sp. nov. (A) Posterior cervical left rib; (B) posterior cervical left rib; (C) posterior cervical right rib (flipped); (D) pectoral left rib; (E) pectoral right rib (flipped); (F) anterior dorsal right rib (flipped); (G) anterior dorsal left rib; (H) middle dorsal right rib (flipped); (I) middle dorsal right rib (flipped); (J) middle dorsal left rib; (K) middle dorsal left rib; (L) posterior dorsal left rib; (M) posterior dorsal right rib (flipped); (N) posterior dorsal left rib; (O) posterior dorsal left rib; (P) posterior dorsal right rib (flipped); (Q) posterior dorsal left rib; (R) sacral left rib; (S) anterior caudal rib; (T) anterior caudal rib. Ribs in dorsal (A and Q–T) and anterior (B–P) views. Asterisks indicate broken bone.

**Cervical ribs:** The anteriormost preserved rib is dorsoventrally-compressed and wing-shaped in dorsal view (Fig. 6A). It is similar to the posterior cervical ribs of other elasmosaurids (e.g., *Albertonectes*; Kubo, Mitchell & Henderson, 2012). Two more posterior cervical ribs are preserved and are strongly arched in anterior view (Figs. 6B and 6C). Their dorsal surfaces have weakly-developed crests that overhang the anterior side, forming a costal groove. The cervical rib heads are gently convex and round to oval.

**Pectoral ribs:** The pectoral ribs are long and straight in anterior view, and have deep costal grooves (Figs. 6D and 6E).

**Dorsal ribs:** The anterior dorsal ribs are long and straight in anterior view, with shallow costal grooves (Figs. 6F–6H). The successive dorsal ribs (Figs. 6I–6Q) become shorter and the costal groove disappears, while the proximodorsal margin forms a prominent knob in anterior view. All of the dorsal rib heads are gently concave, and oval to round.

**Sacral ribs:** The sacral ribs are straight (Figs. 4O and 6R) and have a robust, convex, oval-shaped head, as well as an anteroposteriorly-elongate distal facet for the proximal end of the ilium.

**Caudal ribs:** The caudal ribs are rectangular in dorsal view, dorsoventrally-compressed, and short. They decrease in length posteriorly along the sequence, and have a convex, oval-shaped head (Figs. 6S and 6T). No caudal chevrons are preserved.

**Figure 7:**
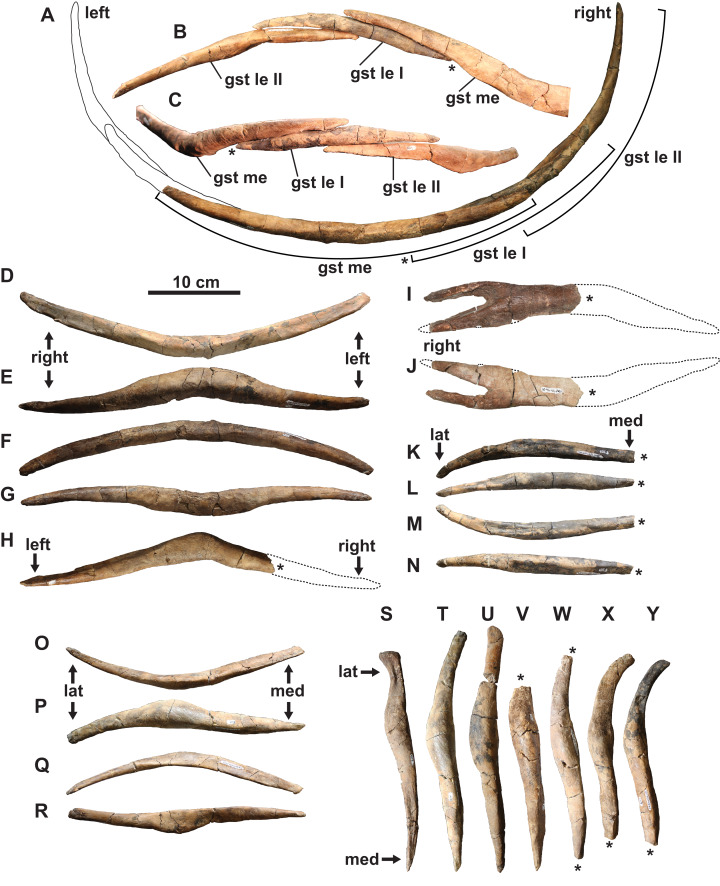
Gastralia of holotype of *Fluvionectes sloanae*, gen. et sp. nov. Reconstructed anterior gastral row in: (A) posterior, (B) anterodorsal and (C) ventral views. Anterior median gastralium in: (D) anterior, (E) ventral, (F) posterior and (G) dorsal views. Posterior median gastralium in (H) dorsal view. Probable anterior median gastralium with two-pronged right side in: (I) ventral and (J) dorsal views. Right anterior first lateral gastralium in: (K) anterior, (L) ventral, (M) posterior and (N) dorsal views. Right anterior second lateral gastralium in: (O) anterior, (P) ventral, (Q) posterior and (R) dorsal views. Second lateral gastralia in ventral view, arranged from anterior (left) to posterior (right): (S) right element, flipped; (T) right element, flipped; (U) left element; (V) left element; (W) left element; (X) right element, flipped and (Y) right element, flipped. Asterisks indicate broken bone. Dotted lines denote estimated reconstructed margin. See “Anatomical Abbreviations”.

**Gastralia:** The disarticulated gastral ribs comprise four median, two first lateral and 10 second lateral elements (Fig. 7). The gastralia are stout and appear to be pachyostotic, as is typical of plesiosaurians, but not as extreme as seen in the cryptoclidid *Tatenectes*, which have a more swollen aspect ratio (O’Keefe et al., 2011). The most complete median element is boomerang-shaped in anterior view with straight rami (Figs. 7A–7G). It is thickest along the midline, and tapers laterally. Much of its ventral surface is covered by parallel striations, which likely represent muscle attachment scarring. The anterior surface of each ramus has a broad, shallow groove which receives the posteromedial surface of each first lateral element. We interpret the median element as likely belonging to the anterior gastral rib sequence, as the angle between the rami is typically smaller in more posterior median elements (Frey et al., 2017). The angle between the rami of another, partial median gastralium (Fig. 7H) is more acute, suggesting that it may have been situated posteriorly. A third, partial median gastralium (Figs. 7I and 7J) appears to have had obtusely angled rami, and is bifurcated on its right side (Fig. 7I). Sato (2002) reported two median gastralia co-ossified at the midline in *Terminonatator*, but the shafts of those two elements are distinct across their entire lengths. Bifurcated gastral ribs otherwise occur in some specimens of the pistosauroid *Corosaurus* (Storrs, 1991), and trifurcated median elements have been reported in an indeterminate elasmosaurid, SDSM 78156 (Martin et al., 2007; Hiller et al., 2017).

The first lateral element is tapered on both ends (Figs. 7A–7C and 7K–7N). The posterior surface is occupied by a groove for the median element, and the anterior surface has a groove to receive the posteromedial surface of the second lateral element. The ventral surface of the first lateral element also has parallel striations.

The second lateral elements are highly variable in form (Figs. 7A–7C and 7S–7Y), but diagnostically each have only one facet on the posteromedial surface. Six of these elements belong to the left side, and four to the right. One of the right elements appears to have been situated posterior to the left elements, suggesting that at least seven rows of gastralia were originally present. This gastral rib count compares with that of the rhomaleosaurid *Macroplata* (Ketchum & Smith, 2010), but exceeds that of the polycotylid *Mauriciosaurus* (six; Frey et al., 2017), the leptocleidid *Nichollsaura* (six; Druckenmiller & Russell, 2008a), and the pliosaurid *Peloneustes* (six; Andrews, 1913). Increased gastral rib rows are known in the pliosaurid *Thalassiodracon* (eight; Smith, 2007), the cryptoclidid *Cryptoclidus oxoniensis* (eight; Brown, 1981), the pliosaurid *Hauffiosaurus zanoni* (eight to 10; Vincent, 2011), the rhomaleosaurid *Atychodracon* (Smith, 2007, 2015), *Plesiosaurus dolichodeirus* (at least nine; Storrs, 1997), the rhomaleosaurid *Meyerasaurus* (10; Smith & Vincent, 2010), *Callawayasaurus* (11; Welles, 1962), the rhomaleosaurid *Rhomaleosaurus thorntoni* (estimated to be 11 or 12; Smith, 2007), and *Pachycostasaurus* (23; Cruickshank, Martill & Noè, 1996).

The second lateral elements (arranged from left to right in Figs. 7S–7Y) include two (Figs. 7T and 7U) that were found articulated (Fig. 2A). The shaft of each is anteroposteriorly narrow towards their medial end, but expand into a rounded shelf at, or slightly lateral to, the midshaft. The lateral end of the anteriormost element (Fig. 7S) is anteroposteriorly expanded, depressed, and bluntly-terminated, with a concave anterior and straight posterior margin. The lateral end is not as expanded in the other elements, and the lateral portion of the shaft becomes more posteriorly-recurved progressing posteriorly along the series.

Our reconstruction of the gastral row (Figs. 7A–7C) places the median (Figs. 7D–7G) and second lateral (Figs. 7O–7R and 7T) elements in the anterior part of the series. The position of the first lateral element (Figs. 7K–7N) is unknown. There is otherwise one lateral pair of gastralia in each row in *Pachycostasaurus* (no median element present; Cruickshank, Martill & Noè, 1996), two in *Fluvionectes sloanae*, *Hauffiosaurus* (Vincent, 2011), SDSM 78156 (Elasmosauridae indet.; Martin et al., 2007; Hiller et al., 2017; O’Gorman et al., 2019) and *Nichollsaura* (Druckenmiller & Russell, 2008a), two to five in *Mauriciosaurus* (Frey et al., 2017), three in *Cryptoclidus oxoniensis* (Brown, 1981), *Macroplata* (Ketchum & Smith, 2010), *Plesiosaurus dolichodeirus* (Storrs, 1997) and three to four in *Meyerasaurus* (Smith & Vincent, 2010).

**Appendicular skeleton**

**Figure 8:**
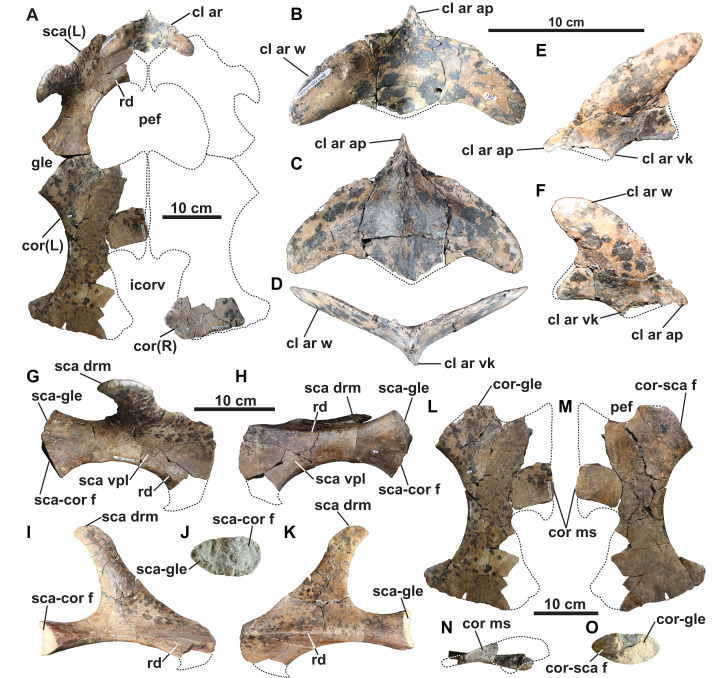
Pectoral girdle of holotype of *Fluvionectes sloanae*, gen. et sp. nov. Girdle in (A) dorsal view. Clavicular arch in: (B) dorsal; (C) ventral; (D) anterior; (E) left ventrolateral and (F) right ventrolateral views. Left scapula in: (G) dorsal; (H) ventral; (I) medial; (J) posterior and (K) lateral views. Left coracoid in: (L) dorsal; (M) ventral; (N) medial and (O) anterolateral views. Dotted lines denote estimated reconstructed margins. See “Anatomical Abbreviations”.

**Clavicular arch:** The small and gracile clavicular arch is missing only the posteromedian margin and part of the anteroventral median keel (Figs. 8A–8K). It consists of both clavicles, and most likely the interclavicle, although no sutures are visible. The clavicular arch is concave dorsally, convex ventrally, dorsoventrally thickest along the midline, and tapers laterally along the clavicular wings. Anteriorly, the midline forms an acute process that projects anteriorly, similar to that of *Morenosaurus* (Welles, 1943: fig. 17), *Thalassomedon* (Welles, 1943: fig. 14), and an elasmosaurid specimen from the Cenomanian Eagle Ford Shale of Texas, TMM 42245-1 (Storrs, 1981: fig. 12). However, it differs from the clavicular arches of *Aphrosaurus* (Welles, 1943: fig. 23b; O’Gorman, 2020: figs. 6a–6c) and *Futabasaurus* (Sato, Hasegawa & Manabe, 2006: fig. 7), which are concave anteriorly or possess a distinct median notch, respectively.

The lateral wings of the clavicles are convex anteriorly, concave posteriorly, and rounded distally, imparting a characteristically ‘boomerang-like’ shape to the clavicular arch in dorsal view (Fig. 8B). The convex anterior margins of the wings are unlike those of *Libonectes*, which were figured by Welles (1949: fig. 2) as having been concave. Sachs & Kear (2017: fig. 6a) illustrated what appears to be part of a clavicular arch in a referred specimen of *Libonectes*, but its morphology is difficult to determine. The wings of the clavicular arch are angled upwards at approximately 23° from the horizontal. Their posterior margins form a deep embayment on each side. This is similar to the condition in *Aphrosaurus* (O’Gorman, 2020), but is unlike that of *Futabasaurus* (Sato, Hasegawa & Manabe, 2006: fig. 7), which has a broad sheet of bone intersecting between the wings and the midline of the arch. The clavicular arches of *Albertonectes* (Kubo, Mitchell & Henderson, 2012: fig. 6), *Cardiocorax* (Araújo et al., 2015: fig. 3), and *Morenosaurus* (Welles, 1943: fig. 17) have relatively straight posterior margins.

The ventral surface has a prominent median keel, but the tip has broken off (Figs. 8C–8F). This keel is thickest in the centre of the element but tapers anteriorly and posteriorly. Similar keels are absent in *Aphrosaurus* (Welles, 1943; O’Gorman, 2020) and *Futabasaurus* (Sato, Hasegawa & Manabe, 2006), or weakly-developed in *Morenosaurus* (Welles, 1943) and prominent in *Albertonectes* (Kubo, Mitchell & Henderson, 2012), *Thalassomedon* (Welles, 1943), TMM 42245-1 (Storrs, 1981), and TTU P 9217 (Chatterjee & Small, 1989). Each wing of the clavicular arch has a smooth ventral surface and appears to lack a distinct facet for the scapula (Fig. 8C). However, the clavicular arch most likely overlapped the scapulae to at least some degree (Fig. 8A).

The interclavicle sutures are at least partly visible in most elasmosaurids, such as *Aphrosaurus* (Welles, 1943; O’Gorman, 2020), *Cardiocorax* (Araújo et al., 2015), *Futabasaurus* (Sato, Hasegawa & Manabe, 2006), *Libonectes* (Welles, 1949), *Morenosaurus* (Welles, 1943), *Styxosaurus browni* (Welles, 1952; Otero, 2016), holotype of ‘*Alzadasaurus pembertoni*’ (Welles & Bump, 1949; Carpenter, 1999), *Thalassomedon* (Welles, 1943), *Wapuskanectes* (Druckenmiller & Russell, 2006), and TMM 42245-1 (Storrs, 1981). On the other hand, the interclavicle is not fused in *Wapuskanectes*. Co-ossification of the interclavicle and clavicles is known in *Futabasaurus* (Sato, Hasegawa & Manabe, 2006) and *Aphrosaurus* (O’Gorman, 2020), both of which were interpreted as represented by osteologically mature specimens. *Albertonectes* (Kubo, Mitchell & Henderson, 2012) and *Callawayasaurus* (Welles, 1962) appear to lack ossified interclavicles.

**Scapula:** The left scapula is missing only its anteromedial end (Figs. 8A and 8G–8K). The shaft is thickened dorsoventrally and narrowed mediolaterally towards its posterior end, but thins and broadens into a flat plate anteriorly. This anterior plate is narrower than in some other elasmosaurids, such as *Cardiocorax* (Araújo et al., 2015), *Vegasaurus* (O’Gorman et al., 2015), and *Zarafasaura* (Lomax & Wahl, 2013). However, the scapula of TMP 1980.031.0001/.0002 (Fig. 5I) approaches the same dimensions as in these latter taxa, suggesting our observed proportional differences may be ontogenetic.

A prominent horizontal ridge extends from the tip of the glenoid facet to the distal edge of the lateral surface of the shaft (Fig. 8K). Such a ridge or keel is present in other elasmosaurids (Welles, 1943; Kubo, Mitchell & Henderson, 2012; Otero, 2016), but extends laterally to form a shelf in leptocleidids (Druckenmiller & Russell, 2008b). Another ridge is present on the dorsal surface of the ventral plate, and extends along the anteromedial edge adjacent to the pectoral fenestra (Figs. 5H, 8A, 8G, and 8I). A comparable ridge is visible in TMP 1980.031.0001/.0002 (Fig. 5I), as well as in *Vegasaurus* (O’Gorman et al., 2015). The comparatively thin flange of bone medial to this ridge is also shared with *Vegasaurus*, and, is in the same position as the posteriorly projecting flange bordering the pectoral fenestra in *Aristonectes quiriquinensis*; interpreted as the acromion tuberosity by Otero et al. (2014).

The orientation of the median coracoid symphysis suggests that the scapulae would have likely contacted each other along the midline (Fig. 8A); this is evident elsewhere in *Aristonectes quiriquiensis* (Otero et al., 2014), *Cardiocorax* (Araújo et al., 2015), and *Zarafasaura* (Lomax & Wahl, 2013), but not in *Callawayasaurus* (Welles, 1962). The dorsal ramus of the scapula projects from the dorsolateral margin of the shaft and tapers to a blunt apex (Figs. 8G–8K). This ramus is dorsoventrally tall and mediolaterally narrow, as in most elasmosaurids, except for *Cardiocorax* (Araújo et al., 2015), in which the dorsal ramus is shorter and is angled at approximately 60° from the horizontal. The rugose coracoid and humerus facets are approximately equal in size (Fig. 8J).

**Coracoids:** The left coracoid is missing its anteromedian and posteromedial margins (Figs. 8A and 8L–8O). The right coracoid preserves only its posterior end (Fig. 8A). The scapular facet of the coracoid is shorter than the glenoid articulation. The anterior margin of the coracoid is strongly concave and supported a pronounced median process. It is uncertain whether the anteromedian process contacted the scapula to form a pectoral bar; however, this is clearly absent in TMP 1980.031.0001/.0002 (Fig. 5I), and thus resembles the condition in most elasmosaurids except for *Elasmosaurus* (Cope, 1868), *Libonectes* (Welles, 1949) and *Wapuskanectes* (Druckenmiller & Russell, 2006). An incomplete pectoral bar is present in *Morenosaurus* (Welles, 1943).

The coracoid preserves part of the symphysial margin, and is dorsoventrally thickened into an interglenoid buttress (Fig. 8N). This forms a distinct ventral process in TMP 1980.031.0001/.0002 (Fig. 5J), which is similar to other elasmosaurids (Druckenmiller & Russell, 2006; Kubo, Mitchell & Henderson, 2012).

The coracoids are separated posteriorly by an intercoracoid vacuity (Figs. 8A, 8L and 8M) as in all elasmosaurids (Brown, 1981), as well as possibly *Leptocleidus superstes* (Kear & Barrett, 2011) and *Brancasaurus* (Sachs, Hornung & Kear, 2016). There are no coracoid fenestrae or notches as occur in *Wapuskanectes* (Druckenmiller & Russell, 2006), together with polycotylids (Williston, 1903; Adams, 1997; Schmeisser McKean & Gillette, 2015) and *L*. *superstes* (Andrews, 1922; Kear & Barrett, 2011). The intercoracoid vacuity is widest anteriorly, and narrows posteriorly, resulting in a cordiform shape, which is unlike the V-shaped opening in *Zarafasaura* (Lomax & Wahl, 2013). The coracoids do not appear to have enclosed the intercoracoid vacuity posteriorly (Figs. 8A, 8L and 8M), as occurs in *Aristonectes quiriquinensis* (Otero et al., 2014), *Cardiocorax* (Araújo et al., 2015), ‘*Alzadasaurus pembertoni*’ (Welles & Bump, 1949; Carpenter, 1999), and *Wapuskanectes* (Druckenmiller & Russell, 2006), although this condition may have changed during ontogeny (see Otero et al., 2014).

The posterior margin of the coracoid is straight, but slopes anterolaterally (Figs. 8A, 8L and 8M). The entire lateral margin is concave, as in most elasmosaurids, but unlike the condition in *Nakonanectes*, in which it is concave anteriorly and abruptly convex posteriorly (Serratos, Druckenmiller & Benson, 2017). Posteriorly, a laterally-projecting posterior cornu extends laterally beyond the glenoid articulation.

**Figure 9:**
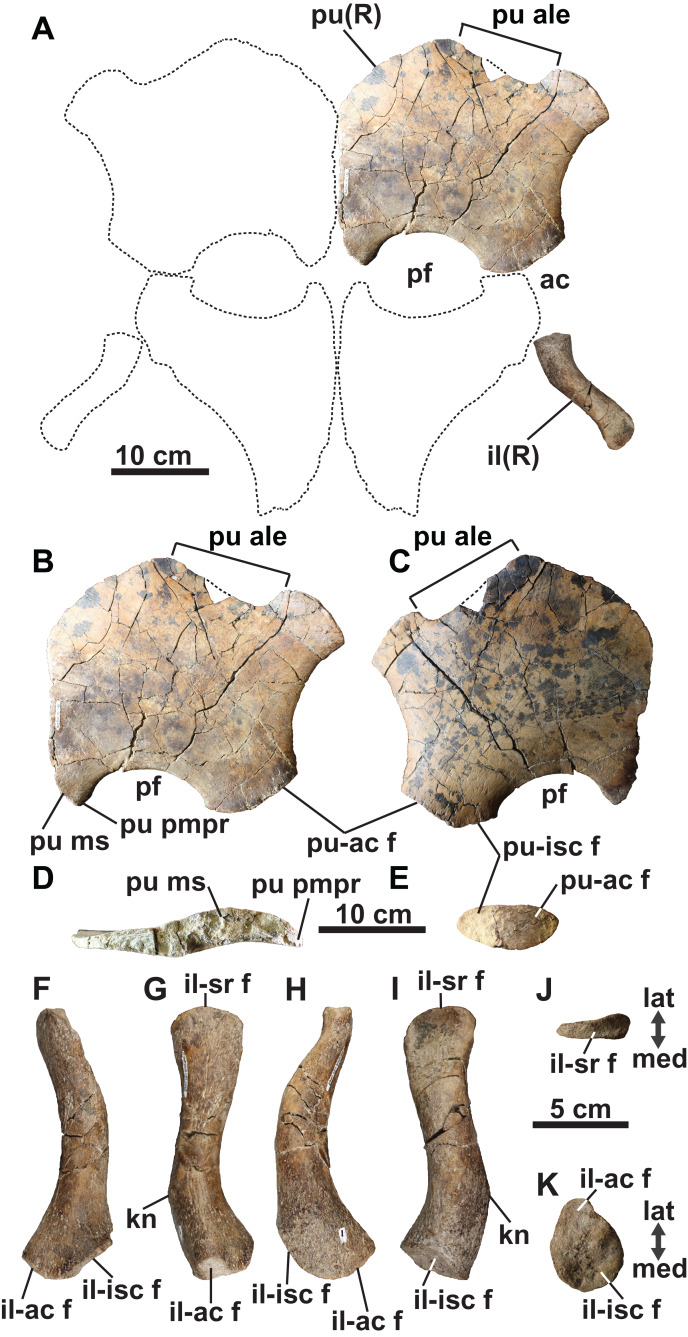
Pelvic girdle of holotype of *Fluvionectes sloanae*, gen. et sp. nov. Girdle in (A) dorsal view. Right pubis in (B) dorsal view; (C) ventral view; (D) posterolateral view and (E) medial view. Right ilium in: (F) anterior; (G) lateral; (H) posterior; (I) medial; (J) proximal and (K) distal views. Dotted lines denote estimated reconstructed margins; ischia reconstruction modified from that of CMN 9895. See “Anatomical Abbreviations”.

**Pubis:** The broad and plate-like right pubis is missing only a small section of its anterolateral margin (Figs. 9A–9E). The median symphysis is thickened along its entire length, and is convex dorsally and concave ventrally. The anteromedial margin of the pubis is convex, whereas the anterolateral margin has a broad shallow embayment. This is absent in elasmosaurids, except for *Callawayasaurus* (Welles, 1962), and some indeterminate specimens from the lower Campanian upper Smoky Hill Chalk Member of the Niobrara Formation in Kansas (see Welles, 1952; Carpenter, 1999), together with polycotylids (e.g., *Dolichorhynchops kirki*, Welles, 1962; *Polycotylus latipinnis*, Carpenter, 1996) and *Brancasaurus* (Wegner, 1914; Sachs, Hornung & Kear, 2016). An anterolateral embayment is likewise present in TMP 1979.008.0006/.0184/.0185 (Fig. 5L) and CMN 9895 (Fig. 5M), and a short nubbin protrudes lateral to the midpoint of the embayment in these specimens, which might have supported a ligamentous attachment for the gastralia.

The anterolateral process of the pubis is gently convex and extends laterally beyond the acetabulum. The lateral margin of the pubis is gently concave, but not deeply excavated as in TMP 1979.008.0006/.0184/.0185 and CMN 9895 (Figs. 5L and 5M). The posterolateral corner of the pubis is thickened and bears rugose acetabular and ischial facets. The acetabular facet is slightly larger than the ischial facet; this is similar to CMN 9895, but differs from TMP 1979.008.0006/.0184/.0185 where facets are of sub-equal size.

The posterior margin of the pubis is dorsoventrally thickened and forms the anterior margin of the pelvic fenestra. The posteromedian process extends to the ischial facet, and has a tapered posterior margin. On the other hand, TMP 1979.008.0006/.0184/.0185 (Fig. 5L) and CMN 9895 (Fig. 5M) have truncated and straight posterior margins that may have supported a pelvic bar like the pubes of *Cardiocorax* (Araújo et al., 2015), *Elasmosaurus* (Cope, 1868) and *Libonectes* (Sachs & Kear, 2017). In *Brancasaurus* (Sachs, Hornung & Kear, 2016), *Futabasaurus* (Sato, Hasegawa & Manabe, 2006), *Kawanectes* (O’Gorman, 2016) and *Libonectes* (Sachs & Kear, 2017), the posteromedian processes enclose a diamond-shaped fenestra, which is absent in *Fluvionectes sloanae*, although a cartilaginous contact might have been present (Sato & Wu, 2006).

**Ilium:** The right ilium (Figs. 9A and 9F–9K) is slender and rod-like with a sub-circular midshaft cross-section as in other elasmosaurids (Kubo, Mitchell & Henderson, 2012). The shaft (Figs. 9G and 9I) is convex posteriorly and concave anteriorly, but less deeply curved than the ilia of *Vegasaurus* (O’Gorman et al., 2015). A knob-like process projects from the posterior surface, about one-third along the length of the shaft, which is a common feature of other elasmosaurids (Hiller, O’Gorman & Otero, 2014; Serratos, Druckenmiller & Benson, 2017; O’Gorman, 2020). The dorsal extremity is anteroposteriorly expanded and mediolaterally compressed with a convex apex and shallow ventrally oriented trough that would have articulated with the sacral ribs (Fig. 9J). The ventral extremity is likewise anteroposteriorly expanded (Fig. 9F) and bears an anterolaterally oriented acetabular facet, offset from a larger and posteromedially oriented ischial facet (Fig. 9K).

**Figure 10:**
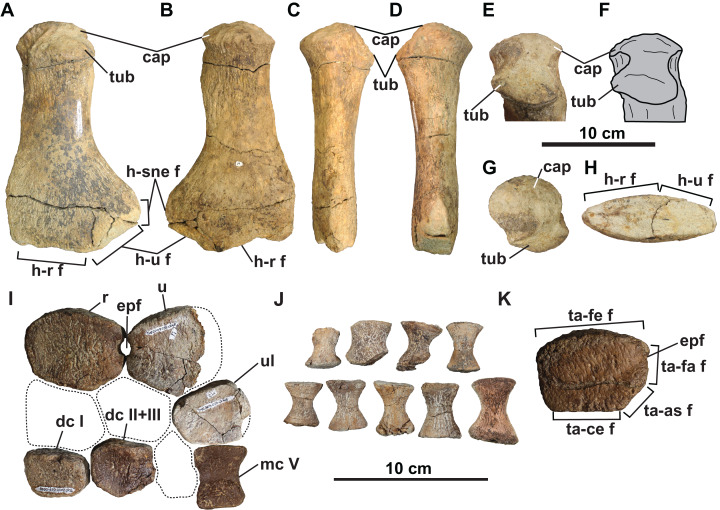
Forelimb (A–J) and hindlimb (K) elements of holotype of *Fluvionectes sloanae*, gen. et sp. nov. Left humerus in: (A) dorsal; (B) ventral; (C) anterior; (D) posterior; proximodorsal (E and F); (G) proximal and (H) distal views. Epipodials, carpals and metacarpal in (I) dorsal or ventral view. Phalanges in (J) dorsal or ventral view. Tibia in (K) dorsal or ventral view. Dotted lines denote estimated reconstructed margins. See “Anatomical Abbreviations”.

**Humerus:** The left humerus has a maximum width/length ratio of 0.57. Its anterior margin is straight proximally, but becomes convex distally. The posterior margin is strongly concave towards the anteroposteriorly expanded distal end, which is most pronounced in CMN 304–309/312–314 (Fig. 5P). The proximal tuberosity is separated from the expansive capitulum, and delimited by a distinct rim (Figs. 10E and 10F). The surfaces of the capitulum and tuberosity are covered by sandstone matrix, but some raised foramina are visible. The ventral surface of the humerus lacks a depression along its anterior margin, which has otherwise been reported in *Kawanectes* (O’Gorman, 2016). Much of the distal articular surface of the humerus is still encased in sandstone. Distally, however, the humerus bears facets for the radius anteriorly and ulna posteriorly, with the ulna facet being larger. An additional small posteriorly deflected facet would have accommodated a supernumerary element, which is also evident in TMP 2009.037.0007 (Fig. 5O) and CMN 304–309/312–314 (Fig. 5P). Supernumerary ossification in the epipodial row are otherwise uncommon among elasmosaurids, having only been reported in *Kawanectes* (O’Gorman, 2016), *Morenosaurus* (Welles, 1943), *Vegasaurus* (O’Gorman et al., 2015), and *Wapuskanectes* (Druckenmiller & Russell, 2006). Supernumerary ossifications in the epipodial to mesopodial rows also occur in *Morenosaurus* (Welles, 1943) and *Nakonanectes* (Serratos, Druckenmiller & Benson, 2017).

**Distal forelimb elements:** Epipodials interpreted as the radius and ulna articulate with the distal end of the left humerus (Fig. 10I). They enclose an epipodial foramen (=spatium interosseum), as in other elasmosaurids (O’Gorman, 2020). The radius is slightly longer anteroposteriorly than proximodistally high, with a height/length ratio of 0.746, which is comparable to *Terminonatator* (0.750) and *Cardiocorax* (0.77) (Araújo et al., 2015). The radius has a long, gently convex proximal facet for the humerus, a short and flat distal facet for the radiale, and a very narrow and flat posterodistal facet for the centrale. The radius contacts the ulna posteriorly via paired facets intersected by the epipodial foramen. The preaxial margin of the radius is straight.

The ulna has a convex proximal facet for the humerus, a posterodistally directed facet for the ulnare, and an anterodistally directed facet for the centrale. The postaxial side of the ulna is not preserved.

The recovered mesopodials include the probable left ulnare, distal carpal I, and co-ossified distal carpal II and III (Fig. 10I). The ulnare has a gently convex posterior margin and flat facets for the ulna anteroproximally, metacarpal V distally, centrale anteriorly, and distal carpal IV anterodistally. Distal carpal I has a straight anterior margin and contacts the radiale proximally, metacarpal I distally, co-ossified distal carpal II and III posteriorly and metacarpal II posterodistally. The co-ossified distal carpal II and III similarly articulates with the centrale proximally, the radiale anteroproximally, distal carpal I anteriorly, metacarpal II anterodistally, metacarpal III, posterodistally and distal carpal IV posteriorly.

A probable metapodial and nine phalanges (Figs. 10I and 10J) were also found but cannot be confidently attributed to either the fore or hind limb. The metapodial resembles metacarpal V of *Albertonectes* (Kubo, Mitchell & Henderson, 2012), and has opposing tapered and faceted surfaces suggesting derivation from either the pre- or postaxial margins. The phalanges (Fig. 10J) are all hourglass-shaped with oval proximal and distal facets.

**Distal hind limb elements:** The only recognisable distal hind limb element is the tibia (Fig. 10K), which is of comparable size to the radius. However, the tibia is more rectangular and appears to have bordered a smaller epipodial foramen. The tibia has a straight anterior margin, convex proximal facet for the femur, and flat contacts with the centrale distally, astragalus posterodistally, and fibula posteriorly.

**Gastroliths**

**Figure 11:**
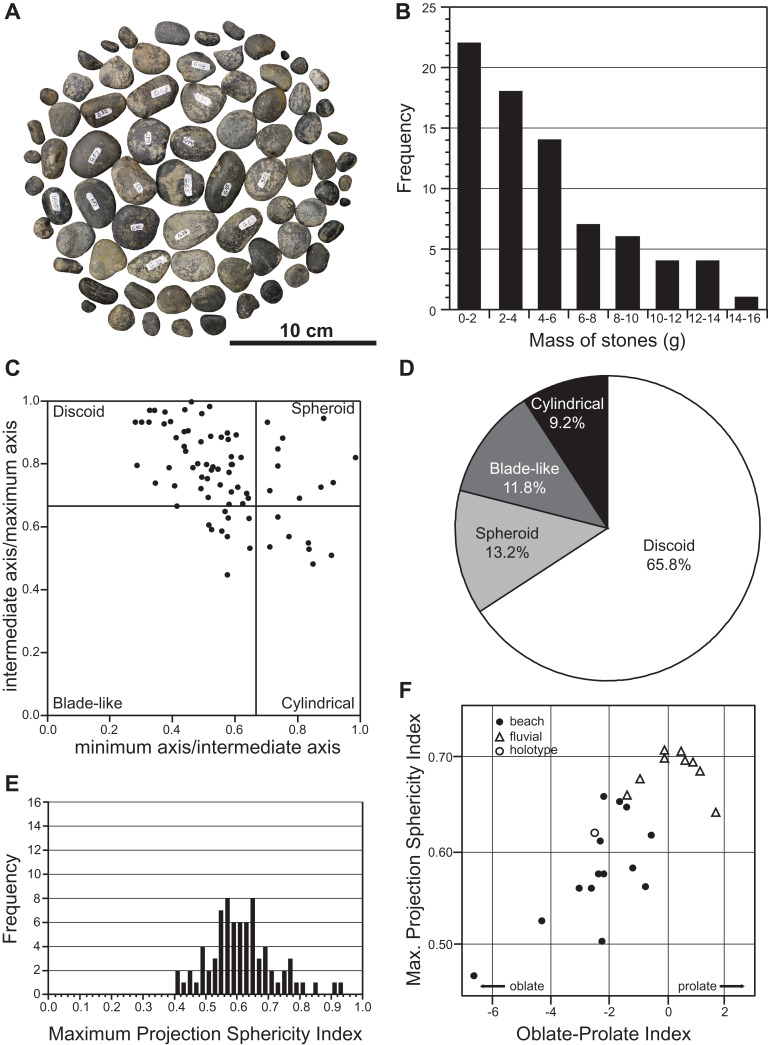
Mass and shape distribution of the 76 gastroliths associated with holotype of *Fluvionectes sloanae*, gen. et sp. nov. (A) Gastroliths (*n* = 76). (B) Histogram showing the mass distribution. (C) Scatter plot showing the shape distribution, formatted after Krumbein (1941). (D) Pie chart showing the percentages of shape types. (E) Histogram showing Maximum Projection Sphericity Index (ψ). (F) Scatter plot of mean Maximum Projection Sphericity (ψ) vs. mean Oblate-Prolate (OP) index values (modified from Dobkins & Folk, 1970: fig. 12; O’Gorman, Olivero & Cabrera, 2012: fig. 7).

A total of 76 smooth, rounded pebbles were found associated with the holotype (Figs. 2A and 11A). Such extra-formational clasts typically occur as strings and thin lenses in the lower DPF (Eberth & Hamblin, 1993). However, we interpreted the pebbles associated with TMP 2009.037.0068/1990.046.0001/.0002 as gastroliths, which frequently occur in association with elasmosaurid remains (Kubo, Mitchell & Henderson, 2012; O’Gorman, Olivero & Cabrera, 2012; O’Gorman et al., 2013; Hiller, O’Gorman & Otero, 2014), as well as more rarely with polycotylids (Sato & Storrs, 2000; Schmeisser & Gillette, 2009; Novas et al., 2015), leptocleidids (Kear, Schroeder & Lee, 2006), and cryptoclidids (Kear, 2006). Gastroliths were concentrated near the pectoral girdle and around the other larger skeletal elements of TMP 2009.037.0068/1990.046.0001/.0002. They are composed of black chert and grey quartzite, with maximum dimensions ranging from 5.1 mm to 38.7 mm. Their masses range from 0.2 g to 15.4 g, with a mean of 4.8 g, and a total combined mass of 361.1 g (Fig. 11B). Most of the gastroliths have irregular gouge marks, suggestive of stone-on-stone contact, as has been described in other plesiosaurian gastric masses (Schmeisser & Gillette, 2009).

We used the discoid, spherical, blade-like, and cylindrical shape criteria of Dobkins & Folk (1970) to categorize the maximum, intermediate, and minimum axes of each gastrolith. The results indicate that approximately two-thirds (65.8%) of the gastroliths are disk-like (Figs. 11C and 11D), consistent with derivation from a beach environment. A smaller proportion (13.2%) of spheroidal gastroliths were likely obtained from fluviatile settings. Further analysis via the Maximum Projection Sphericity Index (ѱ = (c^2^/b × a)^1/3^) and Oblate–Prolate Index ((OP = (10/(c/a)) × ((a − b)/(a − c) − 0.5)), where “a”, “b” and “c” represent the maximum, intermediate, and minimum axes) of Dobkins & Folk (1970) yields mean values of 0.62 (standard deviation = 0.11; Fig. 11E) and −2.54 (standard deviation = 6.12), respectively. These values indicate low-wave-energy beach environments (compare with Dobkins & Folk, 1970) (Fig. 11F) and is generally lower than previous estimates from other plesiosaurian gastrolith assemblages (see Darby & Ojakangas, 1980; Everhart, 2000; Cerda & Salgado, 2008; Schmeisser & Gillette, 2009; O’Gorman, Olivero & Cabrera, 2012; O’Gorman et al., 2013), which are otherwise more applicable to fluvial settings.

**Description of TMP 2009.037.0007**

**Figure 12:**
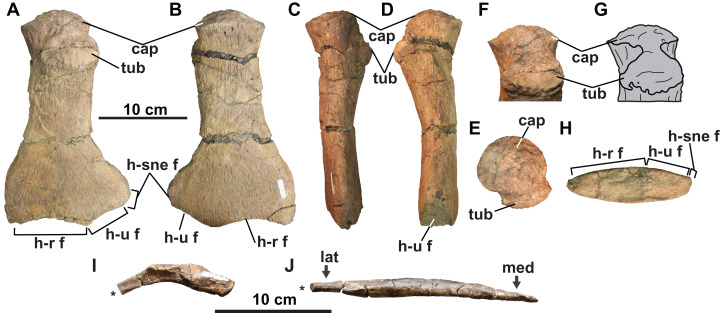
Skeletal elements of TMP 2009.037.0007, referred specimen of *Fluvionectes sloanae*, gen. et sp. nov. Left humerus in: (A) dorsal; (B) ventral; (C) anterior; (D) posterior; proximodorsal (E and F); (G) proximal and (H) distal views. Right posterior cervical rib in (I) anterior view. Right first lateral gastralium in (J) dorsal view. Asterisks indicate broken bone. See “Anatomical Abbreviations”.

TMP 2009.037.0007 consists of a humerus and a partial rib and gastralium. The left humerus (Figs. 12A–12H) resembles that of the holotype of *Fluvionectes sloanae*, but is slightly larger, and has a slightly larger width/length ratio of 0.61. The capitulum and tuberosity are almost completely separated from each other, connected only by a narrow isthmus. The facet for the radius is longer than that of the ulna. The right posterior cervical rib and right first lateral gastralium (Figs. 12I and 12J) compare well with those of the holotype (Figs. 6C and 7N, respectively).

**Phylogenetic analysis**

We conducted a phylogenetic analysis in order to assess the evolutionary relationships of *Fluvionectes sloanae* among other elasmosaurids. We used the dataset of Sachs, Lindgren & Kear (2018), which is in turn based on the matrix of Serratos, Druckenmiller & Benson (2017), but includes *Lagenanectes* (Sachs, Hornung & Kear, 2017). We also added recent matrix updates for *Libonectes* (Sachs & Kear, 2017; scored as a hypodigm to include synonymous taxon *Libonectes atlasense*), *Brancasaurus* (Sachs, Hornung & Kear, 2016; scored as a hypodigm to include synonymous taxon *Gronausaurus*), and *Styxosaurus snowii* (Sachs, Lindgren & Kear, 2018; restricted to the holotype). Our final matrix included 270 characters and 92 taxa with 23 elasmosaurids. The character-taxon matrix was assembled in Mesquite v.3.51 (Maddison & Maddison, 2018), and is provided as a NEXUS file in Supplemental File 3. We conducted the analysis in PAUP*4.0a165 (Swofford, 2002). All characters were equally weighted and all character states were treated as unordered. A heuristic search was conducted with 10,000 replicates with 100 trees saved per replication and using tree bisection and reconnection (TBR) branch swapping. We started with random trees, with a random seed of 0. Bootstrap values were also obtained with 1,000 replicates.

**Figure 13:**
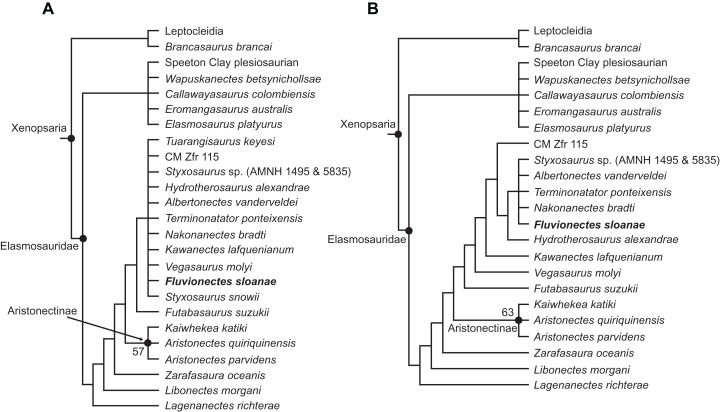
Strict reduced consensus topologies of Elasmosauridae based on analysis of the full matrix after exclusion of wildcard taxa from the set of most parsimonious trees (MPTs). (A) All taxa included (strict consensus of 4,200 MPTs of 1,458 steps; consistency index (CI) = 0.27; retention index (RI) = 0.68); (B) exclusion of the wildcard taxa *Styxosaurus snowii* and *Tuarangisaurus keyesi* (strict consensus of 3593 MPTs of 1445 steps; CI = 0.27; RI = 0.68). Bootstrap values above 50% are given for each node.

Our analysis resulted in 4,200 most parsimonious trees (MPTs) of 1,458 steps. In the resulting strict consensus tree (Fig. 13A; consistency index (CI) = 0.27; retention index (RI) = 0.68), elasmosaurids form a monophyletic sister clade to Leptocleidia (comprising Leptocleididae and Polycotylidae) and *Brancasaurus*. The recovery of *Brancasaurus* as a basal sister to Leptocleidia is consistent with Sachs, Lindgren & Kear (2018: figs. 5c and 5d). Elasmosauridae comprised: (1) an unresolved clade comprising the “Speeton Clay plesiosaurian”, *Wapuskanectes*, *Callawayasaurus*, *Eromangasaurus* and *Elasmosaurus*, which is usually grouped among other Late Cretaceous taxa (Otero, 2016; Sachs, Lindgren & Kear, 2018; O’Gorman, 2020); and (2) a more inclusive polytomy incorporating *Fluvionectes sloanae* and all other elasmosaurids + aristonectines (= *Kaiwhekea*, *Aristonectes quiriquinensis* and *Aristonectes parvidens*, sensu Otero, Soto-Acuña & Rubilar-Rogers, 2012). The recovery of *Lagenanectes* as the basal-most elasmosaurid is consistent with Sachs, Hornung & Kear (2017), but contrasts with Sachs, Lindgren & Kear (2018), who returned *Lagenanectes* as sister to Leptocleidia.

The poor resolution among elasmosaurids is driven by the wildcards *Styxosaurus snowii* and *Tuarangisaurus*, which were identified by comparing the strict and Adams consensus trees. In order to improve resolution, we implemented a strict reduced consensus approach (Wilkinson, 2003) by pruning these wildcards from the MPTs prior to re-computing a strict consensus. This yielded 3593 MPTs of 1,445 steps (Fig. 13B; CI = 0.27; RI = 0.68; see Fig. S1.2 for strict reduced consensus tree of Plesiosauria), and placed *Fluvionectes sloanae* in an unresolved grouping with *Styxosaurus* sp. (AMNH 1495 & 5835), *Albertonectes*, *Terminonatator* and *Nakonanectes*. These taxa all derive from Campanian–Maastrichtian strata in the Western Interior Basin, and are united by the presence of: (1) the dorsal portion of squamosal posterior margin being inflected abruptly anterodorsally (61.1; also present in *Callawayasaurus* and *Eromangasaurus*, among elasmosaurids; approximately straight in *Terminonatator*); (2) the coronoid eminence formed mainly by the dentary (115:1; also present in *Callawayasaurus*, *Eromangasaurus*, and *Styxosaurus snowii*, among elasmosaurids); (3) a ‘heterodont’ (=anisodont sensu Kear et al., 2017) maxillary dentition (133:1; also present in *Lagenanectes*, *Libonectes*, and *Styxosaurus snowii*, among elasmosaurids); (4) the shape of anterior to middle cervical centra being substantially longer than high (153.3; also present in *Elasmosaurus*, among elasmosaurids (not as long in other elasmosaurids); the centra being approximately as long as high in *Nakonanectes*); (5) the ventral surfaces of the caudal centra bearing paired foramina subcentralia (191:0; also present in *Lagenanectes* (pair and single foramen) and *Libonectes*, among elasmosaurids; pair and single foramen in *Fluvionectes sloanae*); (6) the dorsal end of the ilium being only slightly anteroposteriorly expanded (224.2; also present in *Elasmosaurus* and Speeton Clay plesiosaurian, among elasmosaurids; expanded in *Albertonectes* and *Styxosaurus* sp.; identified as present in *Kawanectes* by O’Gorman, 2020); (7) anterolateral cornua on the pubis extending farther laterally than the acetabulum (230:1; also present in Speeton Clay plesiosaurian, among elasmosaurids); (8) a small postaxial accessory ossicle present on limb (232:1; small ossicle also present in *Aristonectes quiriquinensis*, among elasmosaurids (larger in some other elasmosaurids); absent in *Albertonectes*); (9) the radius to tibia length ratio of the epipodials being between 0.9 and 1.09 (242:1; also present in *Kaiwhekea*, *Libonectes*, and Speeton Clay plesiosaurian, among elasmosaurids; less than 0.89 in *Styxosaurus* sp., and between 1.1 and 1.3 in *Albertonectes*); (10) the femoral length to width ratio being between 1.55 and 2.00, showing more elongate proportions than those of other elasmosaurids (251:2; also present in *Futabasaurus*, among elasmosaurids); and (11) the mediolateral width of the tibia 10% greater than that of the fibula (265:0; unique among elasmosaurids).

*Fluvionectes sloanae* can be distinguished from *Styxosaurus* sp., *Albertonectes*, *Terminonatator*, and *Nakonanectes* by its ventrally-notched anterior dorsal vertebrae and postaxial supernumerary epipodial facet on the humerus. *Fluvionectes sloanae* also has 22 dorsal vertebrae, whereas *Albertonectes* has 16 and *Styxosaurus* sp. has 19 (unknown in *Nakonanectes* and count uncertain in *Terminonatator*). *Fluvionectes sloanae* additionally has five sacral vertebrae, whereas *Styxosaurus* sp. has three and *Terminonatator* has four (*Albertonectes* has five; unknown in *Nakonanectes*). The clavicular arch of *Fluvionectes sloanae* is likewise distinct from that of *Albertonectes*, as the former has an embayed (not straight, as in the latter) posterior margin (arch not preserved in *Styxosaurus* sp., *Terminonatator*, and *Nakonanectes*). The anterolateral embayment of the pubis in *Fluvionectes sloanae* is absent in *Albertonectes* (M. T. Mitchell, 2020, personal observation) and *Styxosaurus* sp. (unknown in *Nakonanectes* and *Terminonatator*). Finally, *Fluvionectes sloanae* differs from *Nakonanectes* in having lateral longitudinal ridges on the cervical vertebrae (as is the case in almost all elasmosaurids); this is evident in the referred specimen CMN 304–309/312–314 (Fig. 5A).

## Discussion

### Ontogenetic status

Based on the criteria of Brown (1981), the holotype of *Fluvionectes sloanae* is osteologically mature based on fusion of the neurocentral sutures throughout many of the preserved vertebrae, co-ossification of the clavicular arch, minimal separation of the capitulum and tuberosity on the humerus, and fully-ossified articular facets on limb elements. However, incomplete fusion of the neurocentral sutures indicates that this individual may have been a young “adult” at the time of death. CMN 304–309/312–314 (Figs. 5A, 5C and 5P), TMP 1998.068.0082 (Fig. 5D), CMN 51829 (Fig. 5E), TMP 1980.031.0001/.0002 (Figs. 5G and 5I), TMP 1979.008.0006/.0184/.0185 (Figs. 5G and 5L), CMN 9895 (Fig. 5M), and TMP 2009.037.0007 (Fig. 5O) are slightly larger (up to 32% longer, compare Fig. 5N (humerus of holotype) with Fig. 5P (humerus of CMN 304–309/312–314)) and more osteologically mature than the holotype, showing fusion of all neurocentral sutures, almost complete separation between the capitulum and tuberosity on the humerus, and advanced development of articular facets on limb elements.

### Body reconstruction and estimation of size

We reconstructed the holotype skeleton of *Fluvionectes sloanae* (Fig. 2B) by situating the first pectoral vertebra dorsal to the anterior half of the scapula based on comparisons with the articulated remains of *Albertonectes* (Kubo, Mitchell & Henderson, 2012), *Hydrotherosaurus* (Welles, 1943), and *Mauriciosaurus* (Frey et al., 2017). The acetabulum was located at the dorsal-sacral vertebral transition as in *Mauriciosaurus* (Frey et al., 2017). Following this restoration, we estimate the complete pectoral to sacral vertebral column to have been 1398 mm in length.

Neck length is variable in elasmosaurids, with cervical vertebral counts ranging from 42 (maximum estimate, but possibly as few as 39) in *Nakonanectes* (Serratos, Druckenmiller & Benson, 2017) to 75 in *Albertonectes* (Sachs, Kear & Everhart, 2013). In *Nakonanectes*, the estimated 2.2 m-long neck represents 39.3% of the maximum estimated postcranial length of 5.3 m, with the maximum body length including the skull being up to 5.6 m (Serratos, Druckenmiller & Benson, 2017). In *Albertonectes*, the 7 m-long neck represents 62.5% of the 11.2 m postcranial skeleton (Kubo, Mitchell & Henderson, 2012). Similarly, caudal vertebral counts include 21 vertebrae in *Thalassomedon*, which equates to 28.3% (1,216 mm) of the post-cervical vertebral length (52 vertebrae; approximately 4299 mm) (Welles, 1943), 33 caudal vertebrae in *Albertonectes*, or 44.5% (1,860 mm) of the post-cervical vertebral length (57 vertebrae; 4,180 mm) (D. Henderson, 2020, personal communication), and 30 caudal vertebrae in *Morenosaurus*, or 45.0% (1,632 mm) of the post-cervical vertebral length (52 vertebrae; approximately 3,627 mm) (Welles, 1943).

Following these proportions, we assume that the 400 mm-long caudal vertebral series of *Fluvionectes sloanae* may have originally been between 552 mm and 1,144 mm in length based on *Thalassomedon* and *Morenosaurus*, respectively. This yields an average of 848 mm, or about 2.2 m for the entire post-cervical vertebral series. Assuming that the post-cervical vertebral series was somewhere between 60.7% and 37.5% of the overall postcranial length, then this would suggest a postcranial length between 3.7 m to 6.0 m, or 4.0 m to 6.3 m with an average of approximately 5.2 m given an estimated skull length of about 300 mm (Fig. 2C). Furthermore, CMN 304–309/312–314 has a humerus that is approximately 32% longer than that of the holotype (Figs. 5N and 5P). Scaling the 5.2 m average body isometrically thus yields an average body size of approximately 6.9 m for the largest known specimen of *Fluvionectes sloanae*.

## Conclusions

*Fluvionectes sloanae* can be distinguished from the elasmosaurid specimens AMNH 5261 and CMN 9454 recovered from the non-marine Horseshoe Canyon Formation of southern Alberta (Sato & Wu, 2006). Brown (1913) considered AMNH 5261 to be an ‘adult’ individual and designated it as the holotype of *Leurospondylus*. However, Sato & Wu (2006) concluded that AMNH 5261 was osteologically immature and thus treated *Leurospondylus* as a nomen dubium. Unlike *Fluvionectes sloanae*, AMNH 5261 lacks ventrally-notched dorsal vertebrae and an anterolateral embayment on the pubis. CMN 9454 also differs from *Fluvionectes sloanae* in lacking ventrally-notched dorsal vertebrae and a postaxial supernumerary epipodial facet on the humerus, as well as possessing a well-developed anteromedian coracoid process and more anteriorly-positioned ventral coracoid process. Sato & Wu (2006) considered CMN 9454 to be a young individual, but more mature than AMNH 5261.

**Figure 14:**
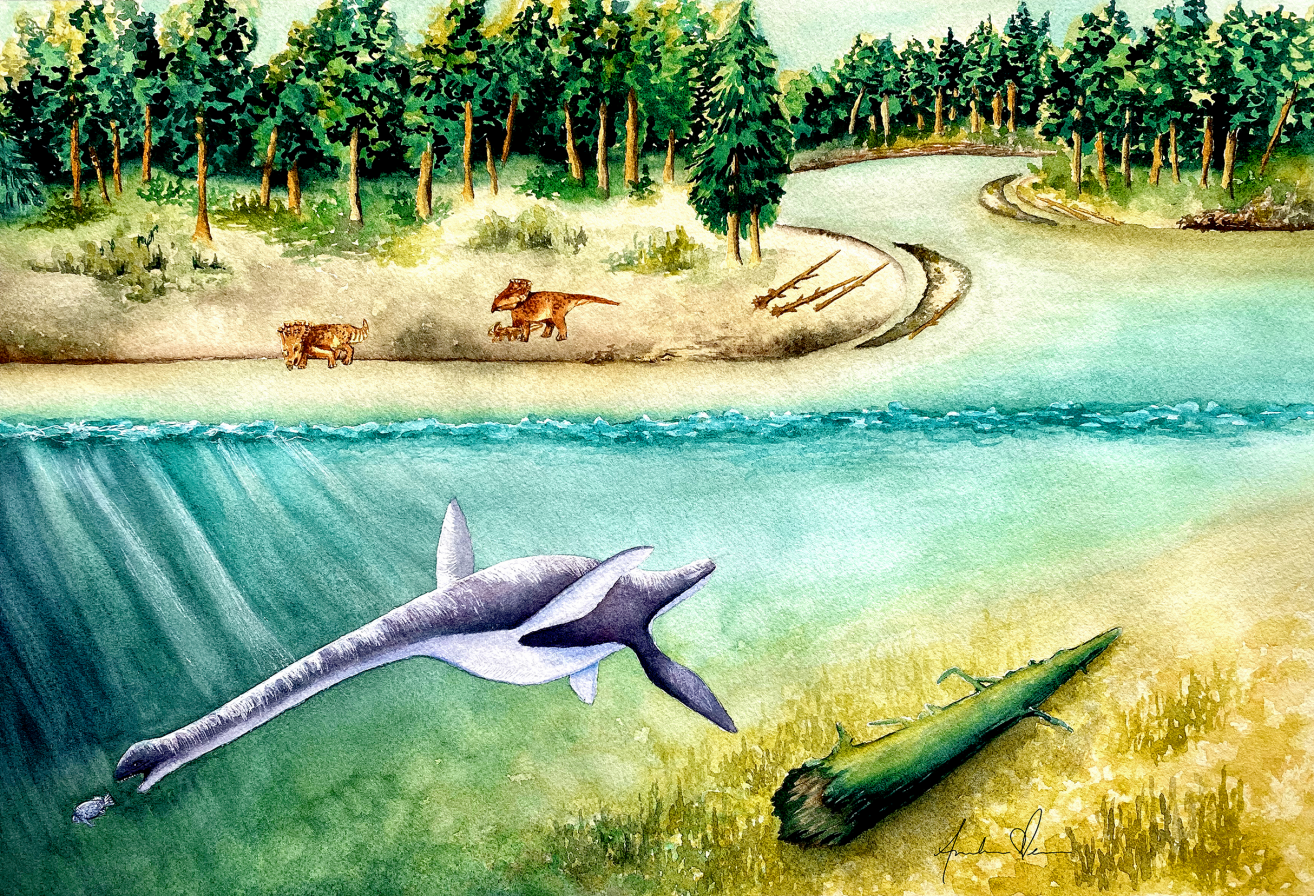
Life reconstruction of *Fluvionectes sloanae*, gen. et sp. nov. Artwork by Andrea Elena Noriega (andreaelena.com).

Both the holotype and TMP 2009.037.0007 were found in estuarine or bay sediments within the DPF. However, TMP 1979.008.0006/.0184/.0185, TMP 1998.068.0082, and TMP 1980.031.0001/.0002 were all collected from fluvial palaeochannel deposits; TMP 1979.008.0006/.0184/.0185 and TMP 1998.068.0082 were collected 37 m above the base of the DPF in DPP (Bonebed 102; approximately 18 m below the base of the LCZ) and TMP 1980.031.0001/.0002 was collected 25.4 m above the base of the DPF in DPP (Quarry 159; approximately 29.6 m below the base of the LCZ) (Fig. 1D; Sato et al., 2005). Given that the lower two-thirds of the DPF, as exposed in DPP, were deposited between 250 and 100 km to the west of the WIS (Eberth, 2005), this indicates that remains of *Fluvionectes sloanae* were buried in an upstream fluvial environment. We therefore suggest that *Fluvionectes* might have at least intermittently inhabited the river systems preserved in the DPF, and represents a rare example of a non-marine plesiosaurian (Supplemental File 1; Fig. 14). It is also plausible that most of the other DPF elasmosaurid fossils also represent *Fluvionectes sloanae*, but their fragmentary nature precludes a definitive taxonomic assignment.

Globally, non-marine plesiosaurian remains have been recovered from Lower Jurassic to Upper Cretaceous lacustrine, fluvial, and estuarine sediments of Argentina, Australia, Canada, China, Germany, and the United Kingdom (Supplemental File 1). These include representatives of almost every plesiosaurian family, Elasmosauridae, Polycotylidae, Leptocleididae, Pliosauridae, and Rhomaleosauridae, and their temporal distribution spans almost the entire plesiosaurian fossil record (Supplemental File 1). Non-marine plesiosaurian remains are usually fragmentary and taxonomically indeterminate (Supplemental File 1). However, some specimens have been identified to species level: *Bishanopliosaurus youngi*, *Bishanopliosaurus zigonensis*, *Brancasaurus brancai*, *Kawanectes lafquenianum*, *Leptocleidus superstes*, *Sinopliosaurus shezisis*, *Sulcusuchus erraini*, *Vectocleidus pastorum*, and *Yuzhoupliosaurus chengjianensis* (Table S1.1; Fig. S1.2). The exceptionally broad phylogenetic and geographic distribution of these taxa (Fig. S1.2) has been cited as evidence for repeated independent radiations into non-marine environments (Kear & Barrett, 2011; Benson et al., 2013a, 2013b).

Non-marine plesiosaurian assemblages generally consist of osteologically immature and/or unusually small-bodied individuals. A similar phenomenon has been documented in mosasaurs, such as *Plioplatecarpus*, which also occurs in estuarine sediments of the LCZ (Caldwell, 2005). Holmes, Caldwell & Cumbaa (1999) additionally reported *Plioplatecarpus* from the St. Mary River Formation at Scabby Butte near Lethbridge, Alberta, which represents an overbank deposit in a flooded coal swamp, located adjacent to a deltaic channel system. Based on these occurrences, *Plioplatecarpus* was considered capable of living in freshwater to estuarine settings. Like modern river dolphins, the physical constrains of non-marine habitats might have imposed a size limit on plesiosaurians, which often incorporate some of the smallest-bodied species within a predominantly large-bodied marine radiation (Cassens et al., 2000; Shostell & Ruiz-García, 2010).

## Supplemental Information

10.7717/peerj.10720/supp-1 Supplemental Information 1 Non-marine plesiosaur occurrences.

10.7717/peerj.10720/supp-2 Supplemental Information 2 Measurements of skeletal elements and gastroliths.

10.7717/peerj.10720/supp-3 Supplemental Information 3 Character-taxon matrix.

## Institutional abbreviations

AMNH: American Museum of Natural History, New York, NY, USA
CM: Canterbury Museum, Christchurch, New Zealand
CMN: Canadian Museum of Nature, Ottawa, ON, Canada
KUVP: Kansas University, Vertebrate Paleontology, Museum of Natural History, Lawrence, KS, USA
NZGS: New Zealand Geological Survey, Lower Hutt, New Zealand
SDSM: South Dakota School of Mines and Technology, Rapid City, SD, USA
TMM: Texas Memorial Museum, Austin, TX, USA
TMP: Royal Tyrrell Museum of Palaeontology, Drumheller, AB, Canada
TTU P: Museum of Texas Tech University, Lubbock, TX, USA

## Anatomical abbreviations

ac: Acetabulum
cap: Capitulum
cauv: Caudal vertebra
chv f: Chevron facet
cl ar: Clavicular arch
cl ar ap: Clavicular arch anterior process
cl ar vk: Clavicular arch ventral keel
cl ar w: Clavicular wing
cor: Coracoid
cor ampr: Anteromedian process of coracoid
cor-gle: Glenoid fossa on coracoid
cor ms: Median symphysis of coracoid
cor-sca f: Facet for scapula on coracoid
cor vpr: Ventral process of coracoid
dc I: Distal carpal I
dc II+III: Distal carpal II and III
dov: Dorsal vertebra
epf: Epipodial foramen
gle: Glenoid fossa
gst le I: Gastralium, 1^st^ lateral element
gst le II: Gastralium, 2^nd^ lateral element
gst me: Gastralium, median element
h-r f: Facet for radius on humerus
h-sne f: Facet for supernumerary epipodial on humerus
h-u f: Facet for ulna on humerus
icorv: Intercoracoid vacuity
il: Ilium
il-ac f: Acetabular facet on ilium
il-isc f: Facet for ischium on ilium
il-sr f: Facet for sacral rib on ilium
isc: Ischium
kn: Knee
lat: Lateral
latlrd: Lateral longitudinal ridge
mc V: Metacarpal V
med: Medial
nsp: Neural spine
pef: Pectoral fenestra
pev: Pectoral vertebra
pf: Pelvic fenestra
poz: Postzygapophysis
prz: Prezygapophysis
pu: Pubis
pu-ac f: Acetabular facet on pubis
pu ale: Anterolateral embayment on pubis
pu-isc f: Facet for ischium on pubis
pu ms: Median symphysis of pubis
pu pmpr: Posteromedian process of pubis
r: Radius
rd: Ridge
ri: Rib(s)
rif: Rib facet
sacv: Sacral vertebra
sca: Scapula
sca-cor f: Facet for coracoid on scapula
sca drm: Dorsal ramus of scapula
sca-gle: Glenoid fossa on scapula
sca vpl: Ventral plate of scapula
ta-as f: Facet for astragalus on tibia
ta-ce f: Facet for centrale on tibia
ta-fa f: Facet for fibula on tibia
ta-fe f: Facet for femur on tibia
trv pr: Transverse process
tub: Tuberosity
u: Ulna
ul: Ulnare
vn: Ventral notch.

## Additional Information and Declarations

### Field Study Permissions

The following information was supplied relating to field study approvals (i.e., approving body and any reference numbers): This study site was accessed under Research and Collection Permit (File No. 3951-E03) issued to David Evans (Royal Ontario Museum), as part of the Southern Alberta Dinosaur Project 2009 field season. The permit was provided by Alberta Culture and Community Spirit Heritage Division and the Royal Tyrrell Museum of Palaeontology.

### Data Availability

The following information was supplied regarding data availability: Raw measurements and character-taxon matrices are available in the Supplemental Files. This article describes plesiosaur specimens currently housed in the collections of the Royal Tyrrell Museum of Palaeontology (TMP) and Canadian Museum of Nature (CMN). The CMN specimens and their corresponding accession numbers (in brackets) are: CMN 51829 (#A-1), CMN 304-309/312-314 (#65), and CMN 9895 (#141). The TMP specimen numbers are the same as their accession numbers, and are: TMP 1979.008.0006, TMP 1979.008.0184, TMP 1979.008.0185, TMP 1980.031.0001, TMP 1980.031.0002, TMP 1990.046.0001, TMP 1990.046.0002, TMP 1998.068.0082, TMP 2009.037.0007, and TMP 2009.037.0068.

### New Species Registration

The following information was supplied regarding the registration of a newly described species: Publication LSID: urn:lsid:zoobank.org:pub:705EFEB6-07D0-4A22-8614-AD436D8AE8DD. *Fluvionectes* gen. nov. LSID: urn:lsid:zoobank.org:act:04CEAEEA-C706-478E-BD51-F802C4DAF746. *Fluvionectes sloanae* sp. nov. LSID: urn:lsid:zoobank.org:act:A7D6D773-1329-40EB-9EE4-C51C96955AD4.
