# Supplementary material for: A new elasmosaurid (Sauropterygia: Plesiosauria) from the non-marine to paralic Dinosaur Park Formation of southern Alberta, Canada
Source: PeerJ. 2021 Feb 11;9:e10720. doi: 10.7717/peerj.10720 (PMC7882142; doi:10.7717/peerj.10720)
Supplement: Supplemental Information 1 [file peerj-09-10720-s001.docx]

**Supplementary File 1. Summary of non-marine plesiosaurian occurrences.**

**Table S1.1.** List of plesiosaurians from non-marine depositional environments, from geologically youngest to oldest.

| **Geological age** | **Formation** | **Locality** | **Taxonomic ID** | **Depositional environment** | **Maturity** | **Body size estimate** | **References** |
| --- | --- | --- | --- | --- | --- | --- | --- |
| **(1) Late Cretaceous** | Thin bed above Strand Fiord Formation, and below Kanguk Formation | Canada (Nunavut, Axel Heiberg Island) | Elasmosauridae indet. | Freshwater | Juvenile | ? | (Vandermark et al., 2006) |
| **(2) Late Cretaceous (Maastrichtian, possibly also uppermost Campanian)** | Horseshoe Canyon Formation | Canada (Alberta, Red Deer River Valley) | Elasmosauridae indet. | Estuarine and alluvial setting | very young to young adult | ? | Brown (1913); Sato & Wu (2006) |
| **(3) Late Campanian or early Maastrichtian** | Allen Formation (middle member) | Argentina (Lago Pellegrini, Contralmirante Cordero, and Salitral de Santa Rosa, Río Negro Province) | *Kawanectes lafquenianum*; *Aristonectes* cf. *parvidens*, Elasmosauridae indet., Polycotylidae indet., Plesiosauria indet. | Estuarine | Adult | 3.8 m for *Kawanectes* | Gasparini & Salgado (2000); O’Gorman et al. (2011, 2013a); O’Gorman (2016) |
| **(4) Late Campanian or early Maastrichtian** | La Colonia Formation (second facies association) | Argentina (Cerro Bayo, Cerro Bosta, Norte de Cerro Bayo 1, and North Cerro Bayo localities, Chubut Province) | *Kawanectes lafquenianum*, Elasmosauridae indet., *Sulcusuchus erraini* | Estuarine, tidal flat, or coastal plain settings | Juvenile, adult | ? | Gasparini & de La Fuente (2000); O’Gorman & Gasparini (2013); O’Gorman et al. (2013b); Gasparini et al. (2015); O’Gorman (2020) |
| **(5) Late Campanian or early Maastrichtian** | Loncoche Formation (Facies Association C) | Argentina (Calmu-Co area, Mendoza Province) | Elasmosauridae indet., Plesiosauroidea indet. | Deltaic plain and channel deposits | ? | ? | Previtera & González Riga (2008) |

**Table S1.1.** Continued.

| **Geological age** | **Formation** | **Locality** | **Taxonomic ID** | **Depositional environment** | **Maturity** | **Body size estimate** | **References** |
| --- | --- | --- | --- | --- | --- | --- | --- |
| **(6) Late Cretaceous (Campanian)** | Dinosaur Park Formation | Canada (Alberta, Red Deer River Valley) | *Fluvionectes sloanae*; Elasmosauridae indet.; Polycotylidae indet. | Fluvial-estuarine | Juvenile to adult | Small | Sato et al. (2005); This Study |
| **(7) Early Cretaceous (early-middle Albian)** | Griman Creek Formation | Australia (Surat Basin, Lightening Ridge, New South Wales and Queensland) | Pliosauroidea indet. | Coastal plain, fluvial/estuarine setting | Small-bodied and/or osteologically immature individuals | Around 2-3 metres | Rich et al. (1989); Kear (2006); Kear et al. (2018) |
| **(8) Early Cretaceous (late Aptian - early Albian)** | Eumeralla Formation | Australia (Otway Basin, southern Victoria) | Pliosauroidea indet. | Meandering to braided river systems in rift valley flood plain | Small-bodied and/or osteologically immature individuals | Around 2-3 metres | Kear (2006) |
| **(9) Early Cretaceous (Aptian/Albian)** | ? | China (Fusui County) | Plesiosauria indet. | Non-marine | ? | ? | Young (1944); Sato et al. (2003) |
| **(10) Early Cretaceous (Aptian)** | Isachsen Formation (Walker Island Member) | Canada (Nunavut, Melville Island) | Polycotylidae indet. and Plesiosauria indet. | Fluvial-deltaic setting | Juvenile to adult | ? | (Vavrek et al., 2014) |
| **(11) Early Cretaceous (late Barremian)** | Vectis Formation | England (Isle of Wight) | *Vectocleidus pastorum* | Lagoonal | ? | ? | Benson et al. (2013) |
| **(12) Early Cretaceous (Barremian)** | Wessex Formation, Wealden Group | England (Tie Pits, Atherfield, Isle of Wight; Compton Bay, Isle of Wight) | Pliosauroidea indet. | Non-marine | ? | ? | Kear & Barrett (2011) |

**Table S1.1.** Continued.

| **Geological age** | **Formation** | **Locality** | **Taxonomic ID** | **Depositional environment** | **Maturity** | **Body size estimate** | **References** |
| --- | --- | --- | --- | --- | --- | --- | --- |
| **(13) Early Cretaceous (Barremian)** | Upper Weald Clay Formation (Weald Clay Group) | England (Sussex, Berwick Brick Pit) | *Leptocleidus superstes* | Lacustrine/lagoonal to fluvial/mudplain | Close to osteological maturity | About 3 m | Andrews (1922); Sato et al. (2003); Kear & Barrett (2011) |
| **(14) Early Cretaceous (Barremian)** | Wessex Formation, Wealden Group | England (Compton Bay, Isle of Wight) | Elasmosauridae indet. | Non-marine | ? | ? | Kear & Barrett (2011) |
| **(15) Early Cretaceous (upper Valanginian)** | Grinstead Clay Member, Hastings Group | England (Cuckfield, Sussex) | Elasmosauridae indet. | Non-marine | ? | ? | Kear & Barrett (2011) |
| **(16) Early Cretaceous (upper Valanginian)** | Turnbridge Wells Sand Formation, Hastings Group | England (St. Leonards, Sussex) | Pliosauroidea indet. | Non-marine | ? | ? | Kear & Barrett (2011) |
| **(17) Early Cretaceous (upper Valanginian)** | Grinstead Clay Member, Hastings Group | England (Hollington, Hastings, Sussex; Cuckfield, Sussex) | Pliosauroidea indet. | Non-marine | ? | ? | Kear & Barrett (2011) |
| **(18) Early Cretaceous (middle Valanginian-Aptian)** | Wonthaggi Formation | Australia (Gippsland Basin, southern Victoria) | Pliosauroidea indet. | Meandering to braided river systems in rift valley flood plain | Small-bodied and/or osteologically immature individuals | Around 2-3 metres | Kear (2006) |

**Table S1.1.** Continued.

| **Geological age** | **Formation** | **Locality** | **Taxonomic ID** | **Depositional environment** | **Maturity** | **Body size estimate** | **References** |
| --- | --- | --- | --- | --- | --- | --- | --- |
| **(19) Early Cretaceous (middle Valanginian)** | Wadhurst Clay Formation, Hastings Group | England (Brenchley, Kent; Hastings, Sussex) | Pliosauroidea indet. | Non-marine | ? | ? | Kear & Barrett (2011) |
| **(20) Early Cretaceous (Berriasian)** | Deister Formation (Obernkirchen Sandstone) | Germany (northwestern Germany) | *Brancasaurus* sp., plesiosauromorph indet., pliosauromorph indet. | Limnic-brackish | Subadult (*Brancasaurus* sp.) | ? | Sachs et al. (2018); Sachs et al. (2016); Koken (1887; 1896) |
| **(21) Early Cretaceous (latest Berriasian)** | Buckeberg Formation | Germany (Gronau, Munsterland, Westphalia) | *Gronausaurus wegneri* | Brackish | Adult | ? | Hampe (2013) |
| **(22) Early Cretaceous (upper Berriasian)** | Bückeberg Group | Germany, near Gronau (Westfalen) | *Brancasaurus brancai* | Lacustrine | Subadult | 3.26 m | Sachs et al. (2016); Wegner (1914) |
| **(23) Latest Jurassic (uppermost Tithonian) to Early Cretaceous (lower Valanginian)** | Purbeck Limestone Group | England (Dorset, southern England) | indeterminate plesiosauroids | Lagoonal-lacustrine | One of the individuals is a juvenile | Small | Kear et al. (2009) |
| **(24) Late Jurassic** | Spilsby Sandstone Formation (Lower Spilsby Sandstone Member) | England (north Lincolnshire, near Nettleton) | Plesiosauria indet., Leptocleididae indet., Pliosauridae indet., Elasmosauridae indet. | Lagoonal setting | Look very immature | small (but immaturity could be contributing largely to this) | Forrest & Oliver (2003) |
| **(25) Middle Jurassic** | Xiashaximiao Formation | China (Zigong, Sichuan) | *Bishanopliosaurus zigonensis* | Fluvial or lacustrine | ? | ? | Gao et al. (2004) |

**Table S1.1.** Continued.

| **Geological age** | **Formation** | **Locality** | **Taxonomic ID** | **Depositional environment** | **Maturity** | **Body size estimate** | **References** |
| --- | --- | --- | --- | --- | --- | --- | --- |
| **(26) Middle Jurassic** | Xintiangou Formation | China (Chenjiang Commune of Chonqing, Sichuan Basin) | *Yuzhoupliosaurus chengjianensis* | Inland lake | ? | 4 m | Zhang (1985) |
| **(27) Middle Jurassic (late Bathonian)** | Xinhe Formation | China (Qingtujing area, Jinchang City, Gansu Province, northwest China) | Pliosauridae indet. | Fluviolacustrine | ? | Small | Gao et al. (2019) |
| **(28) Early or Middle Jurassic (late Toarcian to Aalenian)** | Xintiangou Formation (upper part) | China (Laojun village, Pu’an town, Yunyang County, Chongqing, Sichuan Basin) | Pliosauroidea indet. | Freshwater lacustrine | Possibly juvenile | ? | Zhang et al. (2020) |
| **(29) Early or Middle Jurassic** | Ziliujing Formation (Dongyuemiao Member) | China (Bishan County, Chonqing Municipality) | *Bishanopliosaurus youngi* | Inland lake | Young individual | ? | Dong (1980); Cruickshank (1997); Sato et al. (2003) |
| **(30) Early Jurassic (Toarcian)** | Evergreen Formation | Australia (SE Queensland) | Plesiosauria indet. | Deltaic to lacustrine | Early ontogeny | ? | Thulborn & Warren (1980); Kear (2012) |
| **(31) Early Jurassic (Sinemurian)** | Razorback beds | Australia (Queensland, near Mount Morgan Copper Mine) | Plesiosauria indet. | High-energy braided river system, grading upwards to lacustrine facies | Sub-adult | 3-4 m | Bartholomai (1966); Molnar (1982); Cruickshank (1997); Kear & Barrett (2011); Kear (2012) |
| **(32) Upper Triassic** | ? | China (central Yunnan) | *Sinopliosaurus shezisis* | ? | ? | ? | Xiao et al. (1991) |
| **(33) Age unknown** | ? | China (south Xinjiang) | ? | Fluvial or lacustrine | ? | ? | Wu (1987) |
| **(34) Age unknown** | ? | China (Sichuan) | Rhomaleosauridae indet. | ? | ? | About 4 m | Peng et al. (2005) |

**Figure S1.1. Geographic distribution of non-marine plesiosaurian localities (yellow stars).** Numbering corresponds with that of Table S1.1, numbered from geologically youngest to oldest. Map Credit: vidiani.com (2011). Licensed under CC BY 3.0 SA.


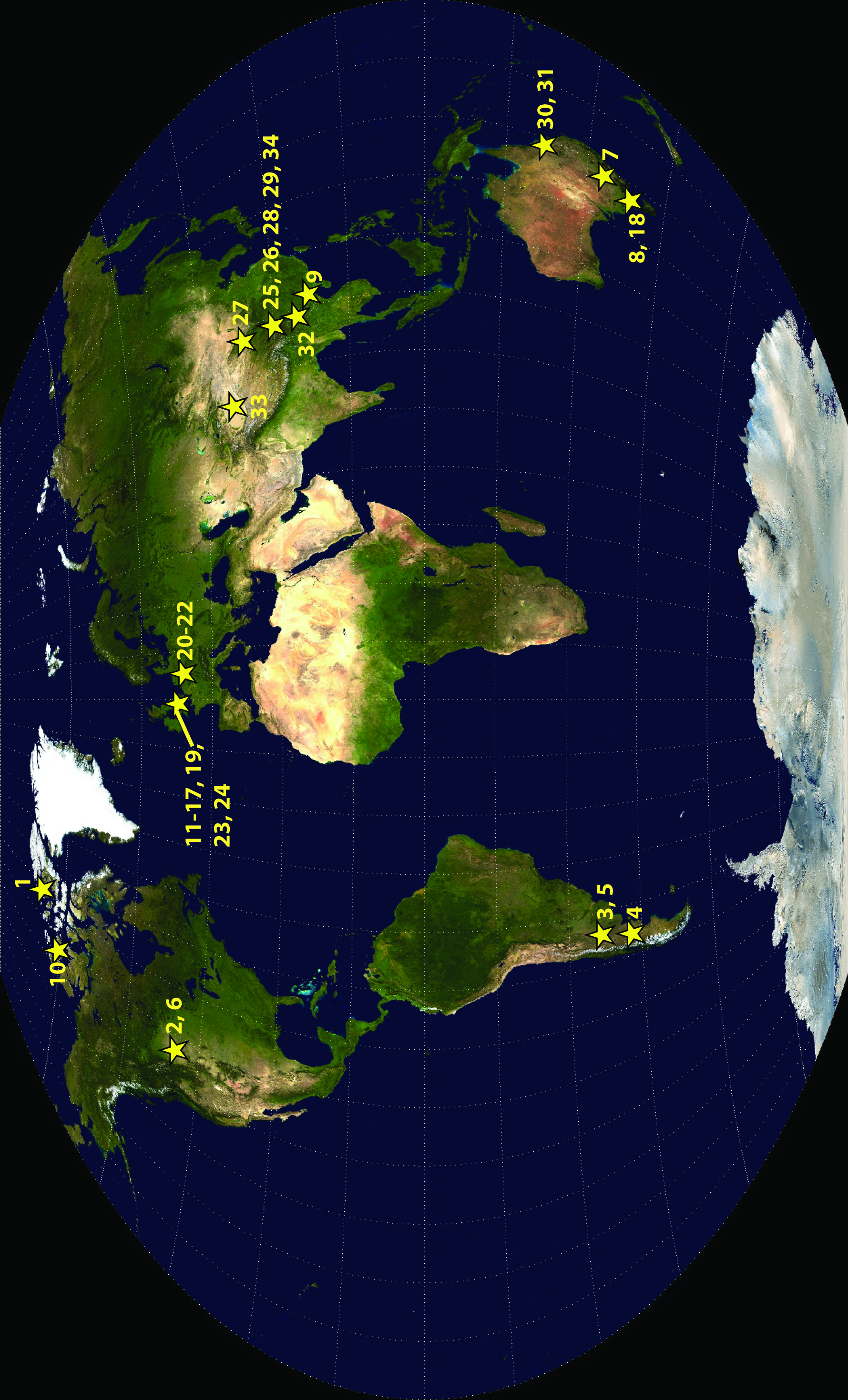


**Figure S1.2. Phylogenetic relationships of non-marine plesiosaurians (asterisks) within Plesiosauria.** Note that the non-marine pliosaurid taxa *Bishanopliosaurus youngi*, *Bishanopliosaurus zigonensis*, *Sinopliosaurus shezisis*, and *Yuzhoupliosaurus chengjianensis*, and the polycotylid taxon *Sulcusuchus erraini* are not included. Most non-marine plesiosaur remains are diagnosable only to the family level. Strict reduced consensus tree of Plesiosauria based on analysis of the full matrix after exclusion of the wildcard taxa *Styxosaurus snowii* and *Tuarangisaurus keyesi* (strict consensus of 3593 most parsimonious trees of 1445 steps; consistency index = 0.27; retention index = 0.68). Bootstrap values are given for each node.


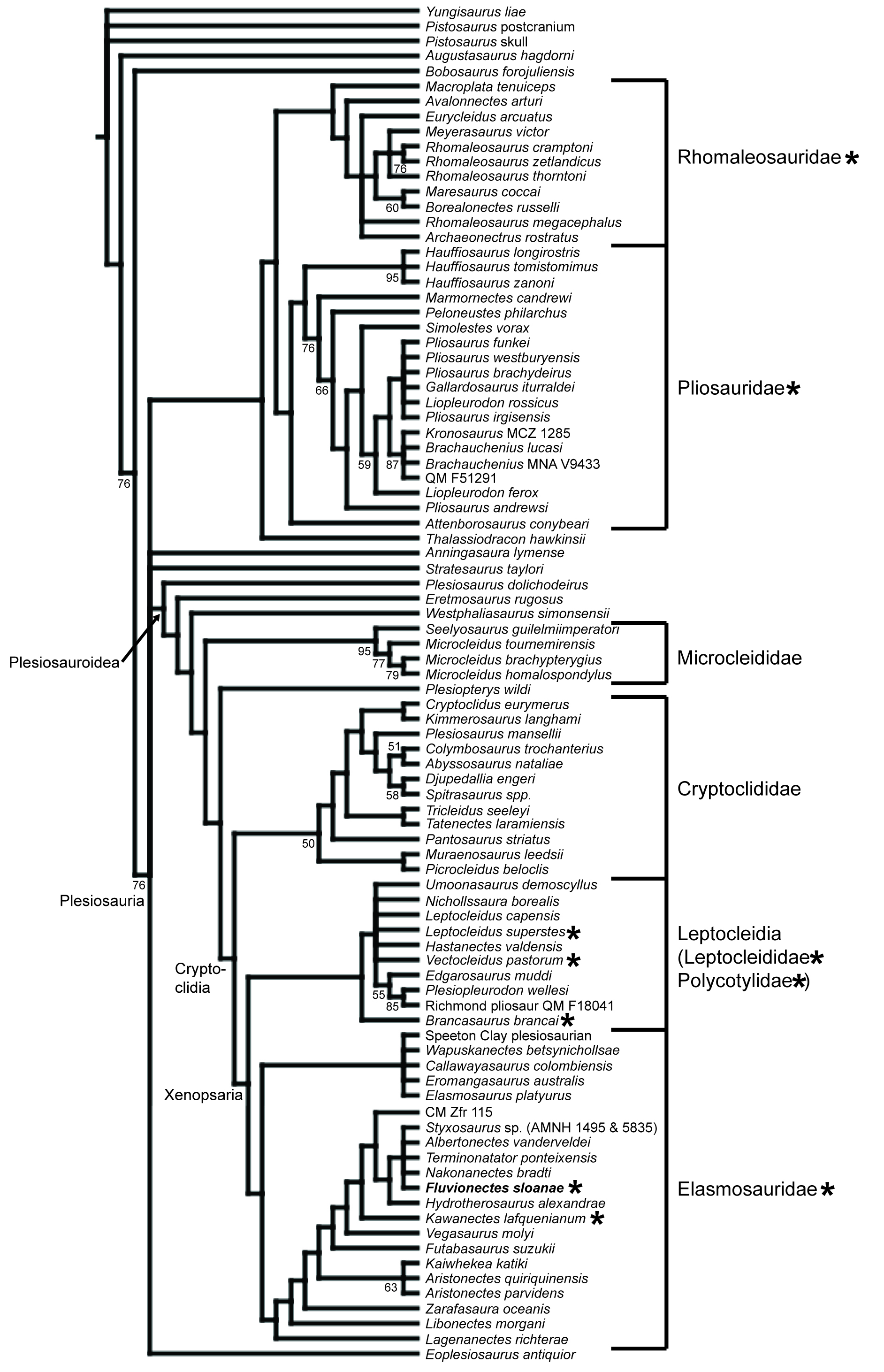


**REFERENCES**

**Andrews CW. 1922.** Description of a new plesiosaur from the Weald Clay of Berwick (Sussex),
 *Quarterly Journal of the Geological Society of London* 78:285–298.
**Bartholomai AL. 1966.** The discovery of plesiosaurian remains in freshwater sediments in
 Queensland, *Australian Journal of Science* 28:437–438.
**Benson BJ, Fitzgerald EMG, Rich TH, Vickers-Rich P. 2013a.** Large freshwater
 plesiosaurian from the Cretaceous (Aptian) of Australia, *Alcheringa: an Australian
 Journal of Palaeontology* 37:456–461. DOI: 10.1080/03115518.2013.772825.
**Benson RBJ, Ketchum HF, Naish D, Turner EL. 2013b.** A new leptocleidid (Sauropterygia,
 Plesiosauria) from the Vectis Formation (early Barremian–early Aptian; Early
 Cretaceous) of the Isle of Wight and the evolution of Leptocleididae, a controversial
 clade, *Journal of Systematic Palaeontology* 11:233–250. DOI: 10.1080/14772019.
 2011.634444.
**Brown B. 1913.** A new plesiosaur, *Leurospondylus*, from the Edmonton Cretaceous of Alberta,
 *Bulletin of the American Museum of Natural History* 32:605–615.
**Cruickshank ARI. 1997.** A Lower Cretaceous pliosauroid from South Africa, *Annals of the
 South African Museum* 105:207–226.
**Dong ZM. 1980.** A new plesiosauria from the Lias of Sichuan Basin, *Vertebrata Palasiatica* 18:
 191–197.
**Forrest R, Oliver N. 2003.** Ichthyosaurs and plesiosaurs from the Lower Spilsby Sandstone
 Member (Upper Jurassic), north Lincolnshire, *Proceedings of the Yorkshire Geological
 Society* 54:269–275. DOI: 10.1144/pygs.54.4.269.
**Gao Y, Ye Y, Jiang S. 2004.** A new species of *Bishanopliosaurus* from Middle Jurassic of
 Zigong, Sichuan, *Vertebrata PalAsiatica* 42:162–165 (in Chinese with English abstract).
**Gao T, Li D-Q, Li LF, Yang JT. 2019.** The first record of a freshwater plesiosaurian from the
 Middle Jurassic of Gansu, NW China, with its implications to the local
 palaeobiogeography, *Journal of Palaeogeography* 8:27. DOI: 10.1186/s42501-019-
 0043-5.
**Gasparini Z, de La Fuente M. 2000.** Tortugas y plesiosaurios de la Formación La Colonia
 (Cretácico superior) de Patagonia, Argentina, *Revista Española de Paleontología* 15:23–
 35.
**Gasparini Z, Salgado L. 2000.** Elasmosáuridos (Plesiosauria) del Cretácico Tardio del norte de
 Patagonia, *Revista Española de Paleontología* 15:13–21.
**Gasparini Z, Sterli J, Parras A, O’Gorman JP, Salgado L, Varela J, Pol D. 2015.** Late
 Cretaceous reptilian biota of the La Colonia Formation, central Patagonia, Argentina:
 occurrences, preservation and paleoenvironments, *Cretaceous Research* 54:154–168.
 DOI: 10.1016/j.cretres.2014.11.010.
**Hampe O. 2013.** The forgotten remains of a leptocleidid plesiosaur (Sauropterygia:
 Plesiosauroidea) from the Early Cretaceous of Gronau (Münsterland, Westphalia,
 Germany), *Paläontol Z* 87:473–491. DOI: 10.1007/s12542-013-0175-3.
**Kear BP. 2006.** Plesiosaur remains from Cretaceous high-latitude non-marine deposits in
 southeastern Australia, *Journal of Vertebrate Paleontology* 26:196–199. DOI: 10.1671/
 0272-4634(2006)26[196:PRFCHN]2.0.CO;2.
**Kear BP. 2012.** A revision of Australia’s Jurassic plesiosaurs, *Palaeontology* 55:1125–1138.
 DOI: 10.1111/j.1475-4983.2012.01183.x.
**Kear BP, Barrett PM. 2011.** Reassessment of the Lower Cretaceous (Barremian) pliosauroid
 *Leptocleidus superstes* Andrews, 1922 and other plesiosaur remains from the nonmarine
 Wealden succession of southern England, *Zoological Journal of the Linnean Society* 161:
 663–691. DOI: 10.1111/j.1096-3642.2010.00648.x.
**Kear BP, Milner AR, Barrett PM. 2009.** Plesiosaur remains from the Jurassic–Cretaceous
 Purbeck Limestone Group of southern England, *Proceedings of the Geologists’
 Association* 120:121–125. DOI: 10.1016/j.pgeola.2009.06.001.
**Kear BP, Fordyce RE, Hiller N, Siversson M. 2018.** A palaeogeographical synthesis of
 Australian Mesozoic marine tetrapods, *Alcheringa: an Australian Journal of
 Palaeontology* 42:461–486. DOI: 10.1080/03115518.2017.1397428.
**Koken E. 1887.** Die Dinosaurier, Crocodiliden und Sauropterygier des norddeutschen Wealden,
 *Geologische und Palaeontologische Abhandlungen* 3:311–420.
**Koken E. 1896.** Die Reptilien des norddeutschen Wealden–Nachtrag, *Geologische* *und
 Palaeontologische Abhandlungen* 7:119–126.
**Molnar RE. 1982.** A catalogue of fossil amphibians and reptiles in Queensland, *Memoirs of the
 Queensland Museum* 20:613–633.
**O’Gorman JP. 2016.** A small body sized non-aristonectine elasmosaurid (Sauropterygia,
 Plesiosauria) from the Late Cretaceous of Patagonia with comments on the relationships
 of the Patagonian and Antarctic elasmosaurids, *Ameghiniana* 53:245–268. DOI:
 10.5710/AMGH.29.11.2015.2928.
**O’Gorman JP. 2020.** First record of *Kawanectes lafquenianum* (Plesiosauria, Elasmosauridae)
 from the La Colonia Formation of Argentina, with comments on the mandibular
 morphology of elasmosaurids, *Alcheringa: an Australian Journal of Palaeontology*
 44:176–193. DOI: 10.1080/03115518.2019.1687754.
**O’Gorman JP, Gasparini Z. 2013.** Revision of *Sulcusuchus erraini* (Sauropterygia,
 Polycotylidae) from the Upper Cretaceous of Patagonia, Argentina, *Alcheringa: an
 Australian Journal of Palaeontology* 37:163–176. DOI: 10.1080/03115518.2013.736788.
**O’Gorman JP, Salgado L, Gasparini Z. 2011.** Plesiosaurios de la Formación Allen
 (Campaniano-Maastrichtiano) en el área del Salitral de Santa Rosa (Provincia de Río
 Negro, Argentina), *Ameghiniana* 48:129–135. DOI: 10.5710/AMGH.v48i1(308).
**O’Gorman JP, Gasparini Z, Salgado L. 2013a.** Postcranial morphology of *Aristonectes*
 (Plesiosauria, Elasmosauridae) from the Upper Cretaceous of Patagonia and Antarctica,
 *Antarctic Science* 25:71–82. DOI: 10.1017/S0954102012000673.
**O’Gorman JP, Salgado L, Varla J, Parras A. 2013b.** Elasmosaurs (Sauropterygia,
 Plesiosauria) from the La Colonia Formation (Campanian–Maastrichtian), Argentina,
 *Alcheringa: an Australian Journal of Palaeontology* 37:259–267. DOI: 10.1080/
 03115518.2013.745250.
**O’Keefe FR, Street HP. 2009.** Osteology of the cryptocleidoid plesiosaur *Tatenectes*

*laramiensis*, with comments on the taxonomic status of the Cimoliasauridae*, Journal of
 Vertebrate Paleontology* 29:48–57. DOI: 10.1671/039.029.0118.
**Peng G, Ye Y, Gao Y, Shu C, Jiang S. 2005.** Jurassic dinosaur faunas in Zigang, pg. 45–
 47. Sichan: Sichuan People’s Publishing House (in Chinese with English abstract).
**Previtera E, González Riga BJ. 2008.** Vertebrados cretácicos de la Formación Loncoche en
 Calmu-Co, Mendoza, Argentina, *Ameghiniana* 45:349–359.
**Rich TH, Rich PV, Wagstaff B, McEwen-Mason J, Douthitt CB, Gregory RT. 1989.** Early
 Cretaceous biota from the northern side of the Australo-Antarctic rift valley. In: Crame
 JA, ed. *Origins and evolution of the Antarctic biota. Special Publication. Geological
 Society of London* 47:121–130.
**Sachs S, Hornung JJ, Kear BP. 2016.** Reappraisal of Europe’s most complete Early Cretaceous
 plesiosaurian: *Brancasaurus brancai* Wegner, 1914 from the “Wealden facies” of
 Germany*, PeerJ* 4:e2813. DOI: 10.7717/peerj.2813.
**Sachs S., Hornung JJ, Lallensack JN, Kear BP. 2018.** First evidence of a large predatory
 plesiosaurian from the Lower Cretaceous non-marine ‘Wealden facies’ deposits of
 northwestern Germany, *Alcheringa: an Australasian Journal of Palaeontology* 42:501–
 508. DOI: 10.1080/03115518.2017.1373150.
**Sato T, Wu X-C. 2006.** Review of plesiosaurians (Reptilia: Sauropterygia) from the Upper
 Cretaceous Horseshoe Canyon Formation in Alberta, Canada, *Paludicola* 5:150–169.
**Sato T, Li C, Wu X-C. 2003.** Restudy of *Bishanopliosaurus youngi* Dong 1980, a freshwater
 plesiosaurian from the Jurassic of Chongqing, *Vertebrata PalAsiatica* 41:17–33.
**Thulborn RA, Warren A. 1980.** Early Jurassic plesiosaurs from Australia, *Nature* 285:224–
 225.
**Vandermark D, Tarduno JA, Brinkman DB. 2006.** Late Cretaceous plesiosaur teeth from
 Axel Heiberg Island, Nunavut, Canada, *Arctic* 59:79–82. DOI: [10.14430/arctic366](https://doi.org/10.14430/arctic366).
**Vavrek MJ, Wilhelm BC, Maxwell EE, Larsson HCE. 2014.** Arctic plesiosaurs from the
 Lower Cretaceous of Melville Island, Nunavut, Canada, *Cretaceous Research* 50:273–
 281. DOI: 10.1016/j.cretres.2014.04.0110195-6671.
**Vidiani.com. 2011.** Detailed satellite map of the world. Website: <[www.vidiani.com/maps/maps
 of_the world/detailed_satellite_map_of_the_world.jpg](http://www.vidiani.com/maps/maps%20%20%09of_the%20world/detailed_satellite_map_of_the_world.jpg)>. Website retrieved July 15, 2019.
**Wegner TH. 1914.** *Brancasaurus brancai* n. g. n. sp., ein Elasmosauride aus dem Wealden

Westfalens. In: Festschrift für Wilhelm Branca zum 70. Geburtstage 1914. Leipzig:

Borntraeger, 235–305.
**Wu S. 1987.** The discovery of Plesiosauria and its significance in south Xinjiang, *Xinjiang
 Geology* 5:105–107 (in Chinese with English abstract).
**Xiao Y-W, Chen K-H, Cao D-B. 1991.** First discovery of *Sinopliosaurus* from Upper Triassic
 of central Yunnan, *Regional Geology of China* 2:180–181 (in Chinese with English
 abstract).

**Young CC. 1944.** On the reptilian remains from Weiyuan, Szechuan, China, *Bulletin of the
 Geological Society of China* 26:187–209.

**Zhang YH. 1985.** A new plesiosaur from Middle Jurassic of Sichuan Basin, *Vertebrata
 Palasiatica* 23:235–240.
**Zhang F, Yu H-D, Xiong C, Wei Z-Y, Peng G-Z, Wei X-F. 2020.** New freshwater
 plesiosaurian materials from the Middle Jurassic Xintiangou Formation of the Sichuan
 Basin, southwestern China, *Journal of Palaeogeography* 9:23. DOI: 10.1186/s42501-
 020-00072-y.
