# Supplementary material for: A new elasmosaurid (Sauropterygia: Plesiosauria) from the non-marine to paralic Dinosaur Park Formation of southern Alberta, Canada
Source: PeerJ. 2021 Feb 11;9:e10720. doi: 10.7717/peerj.10720 (PMC7882142; doi:10.7717/peerj.10720)
Supplement: Supplemental Information 2 [file peerj-09-10720-s002.docx]

**Supplementary File 2: Measurements of skeletal elements and gastroliths.

Table S2.1. List of measurements and notes of vertebral centra of holotype of *Fluvionectes sloanae* (TMP 2009.037.0068/1990.046.0001/.0002).** Centra are numbered in order of their probable anatomical sequence; centra numbers in brackets represent the numbers that these centra are labelled as in collections; “TMP 1990.046.0001” and “TMP 1990.046.0002” in brackets denotes vertebrae that are catalogued under these specimen numbers (all others are catalogued under TMP 2009.037.0068).

| **Centrum** | **Width (mm)** | **Height (mm)** | **Length (mm)** | **Foramina subcentralia** | **Ventral notch** | **Chevron facets, anterior (ant.), posterior (pos.)** | **Neural arch fused** |
| --- | --- | --- | --- | --- | --- | --- | --- |
| **Cervical 1** (TMP 1990.046.0002) | 52.50 | 34.00 | 36.5 | 2 | yes | no | yes |
| **Cervical 2** (no #) | 58.97 | 34.24 | 40.86 | 2 | yes | no | yes |
| **Pectoral 1** (no #) | 63.84 | 41.47 | 41.22 | 2 | no | no | yes |
| **Pectoral 2** (no #) | 64.64 | 41.03 | 40.70 | 2 | no | no | yes |
| **Pectoral 3** ("VD12") | 61.31 | 38.29 | 44.13 | 2 | yes | no | yes |
| **Dorsal 1** ("VD16") | 64.23 | 36.92 | 44.72 | several | yes | no | yes |
| **Dorsal 2** ("VD15") | 64.15 | 40.43 | 45.94 | 2 | yes | no | yes |
| **Dorsal 3** ("VD13") | 62.07 | 41.18 | 49.77 | several | yes | no | yes |
| **Dorsal 4** ("VD11") | 61.96 | 41.42 | 50.30 | several | yes | no | no |
| **Dorsal 5** ("VD19") | 60.87 | 45.55 | 50.71 | 2 | yes | no | yes |
| **Dorsal 6** ("VD14") | 60.22 | 46.30 | 51.82 | 2 | yes | no | no |
| **Dorsal 7** ("VD17") | 60.09 | 47.25 | 50.65 | 2 | yes | no | no |
| **Dorsal 8** ("VD18") | 60.74 | 46.76 | 51.46 | ? | yes | no | no |
| **Dorsal 9** (no #) | 65.34 | 49.55 | 52.99 | several | no | no | yes |
| **Dorsal 10** ("VD8") | 60.67 | 46.82 | 50.10 | several | no | no | no |
| **Dorsal 11** ("VD7") | 60.4 | 47.55 | 51.25 | several | no | no | no |
| **Dorsal 12** ("VD5") | 60.83 | 46.92 | 51.42 | several | no | no | no |
| **Dorsal 13** ("VD6") | 61.29 | 47.46 | 49.16 | 2 | no | no | no |
| **Dorsal 14** ("VD9") | 59.36 | 44.00 | 49.49 | several | no | no | no |
| **Dorsal 15** (no #) | 60.83 | 44.60 | 47.44 | 2 | no | no | yes |
| **Dorsal 16** (TMP 1990.046.0001) | 63.00 | 46.00 | 52.00 | ? | no | no | yes |
| **Dorsal 17** (TMP 1990.046.0001) | 59.50 | 41.00 | 44.00 | ? | no | no | yes |
| **Dorsal 18** ("VD4") | 59.16 | 40.32 | 44.09 | ? | no | no | no |
| **Dorsal 19** ("VD10") | 60.01 | 41.86 | 44.28 | ? | no | no | no |
| **Dorsal 20** ("VD2") | 58.88 | 39.50 | 44.90 | 2 | no | no | no |
| **Dorsal 21** ("VD1") | 57.90 | 39.13 | 45.21 | 2 | no | no | no |
| **Dorsal 22** ("VCA2") | 56.66 | 39.03 | 43.38 | several | yes | no | no |
| **Sacral 1** ("VD3") | 56.80 | 40.52 | 41.57 | 4 | no | no | no |
| **Sacral 2** (TMP 1990.046.0001) | 58.00 | 43.00 | 44.00 | several | no | no | yes |
| **Sacral 3** ("VS2") | 58.91 | 39.24 | 43.00 | several | yes | no | no |
| **Sacral 4** ("VS1") | 59.25 | 41.3 | 40.16 | 1 | yes | no | no |
| **Sacral 5** (no #) | 64.05 | 43.40 | 37.93 | 1 | ? | no | yes |
| **Caudal 1** (TMP 1990.046.0001) | 59.00 | 43.00 | 36.00 | 2 | no | no | yes |
| **Caudal 2** (“VS3”) | 59.67 | 41.19 | 35.88 | 2 | no | no | no |
| **Caudal 3** ("VCA8") | 62.36 | 42.85 | 33.93 | 2 | no | no | yes |
| **Caudal 4** ("VCA7") | 57.97 | 40.68 | 33.91 | 1 | no | pos. | no |
| **Caudal 5** ("VCA1") | 59.02 | 40.69 | 33.67 | 1 | no | pos. | no |
| **Caudal 6** ("VCA6") | 59.21 | 40.09 | 34.25 | 1 | no | pos. | no |
| **Caudal 7** ("VCA5") | 59.29 | 39.58 | 33.17 | 2 | no | pos. | no |
| **Caudal 8** (TMP 1990.046.0001) | 56.00 | 41.50 | 32.50 | 1 | no | pos. | yes |
| **Caudal 9** ("VCA4") | 58.22 | 39.17 | 32.61 | 1 | no | pos. | no |
| **Caudal 10** ("VCA3") | 57.64 | 39.60 | 32.79 | 1 | no | ant., pos. | no |
| **Caudal 11** (no #) | 57.45 | 39.42 | 32.28 | 2 | no | ant., pos. | no |
| **Caudal 12** ("VCA9") | 59.11 | 39.31 | 29.37 | 1 | no | ant., pos. | yes |

**Figure S2.1.** **Line drawings of select postcranial skeletal elements of elasmosaurid specimens discussed in text, and their measurements; see Table S2.1 for measurements.** Elements of holotype of *Fluvionectes sloanae* (A–F): **A)** pectoral girdle in dorsal view; **B)** left forelimb elements in dorsal view; **C)** left scapula in ventrolateral view; **D)** right pubis in dorsal view; **E)** right ilium in posterior (left) and medial (right) views; and **F)** tibia in dorsal or ventral view. Left humerus of TMP 2009.037.0007 in **G)** dorsal view. Left pubis of TMP 1979.008.0006/.0184/.0185 in **H)** dorsal view. Right pubis of CMN 9895 in **I)** dorsal view. Left scapula and coracoid of TMP 1980.031.0001/.0002 in **J)** dorsal view. Right humerus of CMN 304–309/312–314 in **K)** ventral view.


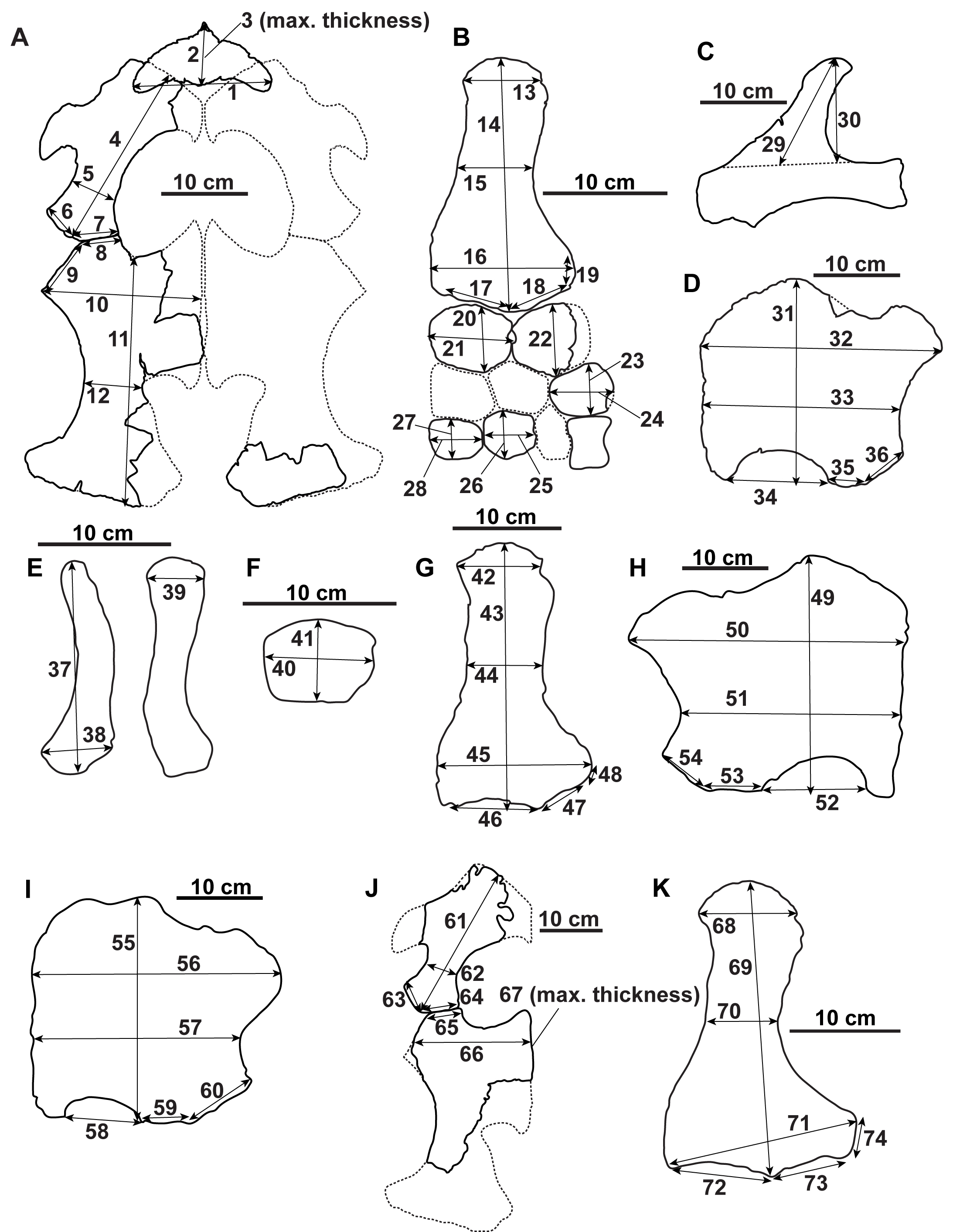


**Table S2.2. List of measurements of holotype of *Fluvionectes sloanae* (1–41), TMP 2009.037.0007 (42–48), TMP 1979.008.0006/.0184/.0185 (49–54), CMN 9895 (55–60), TMP 1980.031.0001/.0002 (61–67), and CMN 304–309/312–314 (68–74) as given in Figure S2.1.**

| **Parameter** | | **Parameter** | | **Parameter** | |
| --- | --- | --- | --- | --- | --- |
| **#** | **Measurement (mm)** | **#** | **Measurement (mm)** | **#** | **Measurement (mm)** |
| **1** | 153.40 | **26** | 37.55 | **51** | 227.86 |
| **2** | 84.67 | **27** | 31.30 | **52** | 110.67 |
| **3** | 22.93 | **28** | 43.92 | **53** | 60.83 |
| **4** | 221.13 | **29** | 141.27 | **54** | 56.15 |
| **5** | 51.55 | **30** | 121.42 | **55** | 232.77 |
| **6** | 45.45 | **31** | 234.40 | **56** | 280.50 |
| **7** | 56.96 | **32** | 262.45 | **57** | 231.81 |
| **8** | 45.83 | **33** | 211.54 | **58** | 84.58 |
| **9** | 63.21 | **34** | 112.85 | **59** | 56.67 |
| **10** | 167.00 | **35** | 41.13 | **60** | 74.13 |
| **11** | 279.45 | **36** | 53.52 | **61** | 261.71 |
| **12** | 65.09 | **37** | 141.78 | **62** | 55.02 |
| **13** | 63.09 | **38** | 48.19 | **63** | 50.96 |
| **14** | 202.49 | **39** | 38.71 | **64** | 66.11 |
| **15** | 61.15 | **40** | 68.08 | **65** | 60.62 |
| **16** | 115.82 | **41** | 50.76 | **66** | 187.71 |
| **17** | 61.83 | **42** | 77.52 | **67** | 70.65 |
| **18** | 50.80 | **43** | 246.71 | **68** | 88.52 |
| **19** | 21.87 | **44** | 75.01 | **69** | 266.02 |
| **20** | 50.21 | **45** | 151.73 | **70** | 67.03 |
| **21** | 66.51 | **46** | 89.50 | **71** | 180.11 |
| **22** | 53.17 | **47** | 62.19 | **72** | 93.60 |
| **23** | 36.67 | **48** | 19.81 | **73** | 70.24 |
| **24** | 47.11 | **49** | 261.33 | **74** | 39.62 |
| **25** | 41.72 | **50** | 288.61 |  |  |

**Table S2.3. List of measurements of gastroliths associated with holotype of *Fluvionectes sloanae*. Gastroliths are numbered as such in collections.**

| **Gastrolith** | **Maximum axis (mm)** | **Intermediate axis (mm)** | **Minimum axis (mm)** | **Mass (g)** |
| --- | --- | --- | --- | --- |
| **1** | 6.43 | 5.68 | 4.27 | 0.2 |
| **2** | 30.73 | 21.24 | 13.66 | 13.8 |
| **3** | 19.93 | 15.99 | 7.71 | 3.7 |
| **4** | 30.66 | 22.64 | 7.86 | 9.3 |
| **5** | 16.64 | 11.52 | 9.29 | 2.7 |
| **6** | 15.83 | 13.92 | 8.04 | 2.2 |
| **7** | 21.25 | 13.82 | 7.87 | 3.8 |
| **8** | 24.7 | 15.48 | 9.99 | 5.8 |
| **9** | 14.69 | 13.28 | 5.83 | 1.7 |
| **10** | 22.21 | 19.73 | 10.33 | 6.6 |
| **11** | 21.03 | 16.44 | 8.64 | 4.6 |
| **12** | 24.78 | 23.15 | 6.99 | 6.3 |
| **13** | 21.3 | 16.47 | 9.59 | 5.5 |
| **14** | 25.99 | 22.95 | 9.44 | 8.7 |
| **15** | 13.88 | 10.3 | 9.41 | 1.8 |
| **16** | 16.91 | 16.87 | 7.79 | 3.4 |
| **17** | 13.39 | 10.64 | 7.83 | 1.5 |
| **18** | 20.82 | 20.08 | 7.57 | 4.6 |
| **19** | 16.42 | 15.96 | 7.04 | 2.7 |
| **20** | 18.25 | 16.31 | 9.85 | 4.8 |
| **21** | 22.46 | 20.18 | 11.65 | 8.1 |
| **22** | 26.75 | 19.32 | 9.48 | 8 |
| **23** | 22.32 | 20.82 | 5.89 | 4.3 |
| **24** | 20.06 | 13.46 | 7.82 | 3.2 |
| **25** | 23.33 | 22.41 | 11.05 | 7.2 |
| **26** | 28.56 | 22.55 | 10.49 | 10.2 |
| **27** | 28.55 | 24.45 | 10.74 | 12.2 |
| **28** | 34.07 | 22.69 | 9.4 | 11.9 |
| **29** | 18.37 | 16.26 | 9.03 | 4.1 |
| **30** | 22.1 | 13.39 | 6.92 | 3.2 |
| **31** | 15.17 | 13.21 | 6.51 | 2.2 |
| **32** | 27.69 | 17.45 | 10.11 | 7.5 |
| **33** | 15.38 | 8.14 | 6.8 | 1.4 |
| **34** | 21 | 17.66 | 7.85 | 4.4 |
| **35** | 11.56 | 10.78 | 7.6 | 1.4 |
| **36** | 18.94 | 14.99 | 7.96 | 3.1 |
| **37** | 10.28 | 7.79 | 3.86 | 0.5 |
| **38** | 20.96 | 19.59 | 7.73 | 5.1 |
| **39** | 10.51 | 7.63 | 6.68 | 0.8 |
| **40** | 29.82 | 27.7 | 10.34 | 12.8 |
| **41** | 22.13 | 20.67 | 6.72 | 5.3 |
| **42** | 38.65 | 20.57 | 13.34 | 15.4 |
| **43** | 22.95 | 18.35 | 10.85 | 6.7 |
| **44** | 28.03 | 21.12 | 10.82 | 10.4 |
| **45** | 8.76 | 6.99 | 3.56 | 0.4 |
| **46** | 10.64 | 10.45 | 5.42 | 0.9 |
| **47** | 18.95 | 13.92 | 7.69 | 2.8 |
| **48** | 17.39 | 8.37 | 7.1 | 1.5 |

**Table S2.3.** Continued.

| **Gastrolith** | **Maximum axis (mm)** | **Intermediate axis (mm)** | **Minimum axis (mm)** | **Mass (g)** |
| --- | --- | --- | --- | --- |
| **49** | 11.1 | 6.31 | 4.88 | 0.5 |
| **50** | 10.89 | 10.31 | 9.11 | 1.5 |
| **51** | 25.13 | 11.26 | 6.5 | 3.2 |
| **52** | 16.13 | 13.3 | 7.82 | 2.5 |
| **53** | 13.82 | 9.3 | 5.8 | 1.2 |
| **54** | 27.15 | 26.37 | 9.04 | 9 |
| **55** | 14.11 | 11.26 | 6.64 | 1.4 |
| **56** | 18.67 | 13.55 | 8.27 | 3.5 |
| **57** | 31.68 | 25.22 | 7.25 | 9.6 |
| **58** | 19.32 | 13.65 | 8.7 | 3.4 |
| **59** | 12.64 | 7.2 | 4.14 | 0.6 |
| **60** | 11.81 | 10.03 | 7.4 | 1.3 |
| **61** | 5.12 | 4.2 | 4.14 | 1 |
| **62** | 14.74 | 10.57 | 7.51 | 1.6 |
| **63** | 14.77 | 7.93 | 5.65 | 1.1 |
| **64** | 15.83 | 13.01 | 8.05 | 2.5 |
| **65** | 30.1 | 29.24 | 9.6 | 13.2 |
| **66** | 32.79 | 19.24 | 10.73 | 11.3 |
| **67** | 31.6 | 18.7 | 9.81 | 9.3 |
| **68** | 23.79 | 18.74 | 7.28 | 4.5 |
| **69** | 23.37 | 16.21 | 8.36 | 5.3 |
| **70** | 27.22 | 19.91 | 8.13 | 6 |
| **71** | 23.38 | 18.33 | 9.98 | 6.4 |
| **72** | 19.52 | 17.66 | 7.95 | 4.1 |
| **73** | 18.15 | 9.26 | 8.4 | 2.2 |
| **74** | 18.41 | 13.1 | 7.73 | 2.8 |
| **75** | 15.66 | 8.6 | 7.18 | 1.6 |
| **unnumbered** | 15.59 | 9.85 | 7.26 | 1.8 |
